# The Combination of Individual Herb of Mi-Jian-Chang-Pu Formula Exerts a Synergistic Effect in the Treatment of Ischemic Stroke in Rats

**DOI:** 10.1155/2022/9365760

**Published:** 2022-10-18

**Authors:** Fangfang Lu, Xiaojuan Su, Jingjing Liu, Rong Zong, Shuqin Ding, Lingling Yang, Jing Liu, Gidion Wilson, Liuyan Li, Youyue Yang, Xiaoying Wang, Weibiao Wang, Xueqin Ma

**Affiliations:** ^1^Department of Pharmaceutical Analysis, School of Pharmacy, Key Laboratory of Hui Ethnic Medicine Modernization, Ministry of Education, Ningxia Medical University, 1160 Shenli Street, Yinchuan 750004, China; ^2^School of Pharmacy, Lanzhou University, 222 Tianshui South Road, Lanzhou 730000, China

## Abstract

Mi-Jian-Chang-Pu formula (MJCPF), composed of *Crocus sativus L*. and *Acorus tatarinowii* Schott, is a well-known TCM for treatment of hemiplegia, facial paralysis as well as language dysfunction caused by stroke both in ancient and modern times. By using pharmacodynamics, pharmacokinetics, and metabolomics, our present study discusses whether the combination of individual herbs or major active components of MJCPF possess synergistic neuroprotective effects against ischemic stroke (IS). 108 adult male Sprague-Dawley rats were randomly and equally divided into 9 groups, including sham group (N, vehicle), middle cerebral artery occlusion (MCAO) model group (M, vehicle), positive group (P, 36 mg/kg/day nimodipine), crocin I (A1, 40 mg/kg/day), *β*-asarone (B1, 15 mg/kg/day), crocin I + *β*-asarone (A1B1, 55 mg/kg/day), *C*. *sativus* (A, 580 mg/kg/day), *A. tatarinowii* (B, 480 mg/kg/day), and *C. sativus* + *A. tatarinowii*, also named MJCPF (AB, 1060 mg/kg/day) groups. All drugs were orally administered to rats once a day for 14 consecutive days. Neurological deficit score, cerebral infarct volume, body weight change, TTC, HE and IHC staining, behavioral evaluation, metabolic profiles, and pharmacokinetic parameters were determined. MCAO led to severe brain damage including large infarct volume, more severe brain tissue injury, and worse neurological function as compared to the sham rats. All treatment groups showed a significant neuroprotective effect on MCAO rats. Furthermore, the pharmacodynamics' results demonstrated that MJCPF had a synergistic effect evidenced by small infarct volume, more regular arrangement of neuronal cells, and more improved neural function, and the levels of inflammatory factors were closer to normality. A total of 53 differential metabolites between MCAO and sham groups were screened by integration of serum and brain metabolisms, all of which were restored at varying degrees in treatment. PCA and PLS-DA analysis showed that the levels of differential metabolites treated with MJCPF were closer to the sham group than the individual herb and single compound alone or A1B1 combination. The pharmacokinetic parameters further verified the above results that MJCPF could synergistically promote drug absorption greater than others. Our integrated pharmacodynamics, metabolomics, and pharmacokinetic approach reveals the synergistic effect of MJCPF on treatment of IS, which powerfully contribute to the understanding of scientific connotation of TMC formula.

## 1. Introduction

Stroke, the world's second-leading death disease, is the main cause of disability worldwide. Its prevalence increases with aging population [1]. As the most populous country in the world, stroke is now the biggest challenge in China. In 2018, the mortality rate of China's cerebrovascular disease was 149.49 per 100,000 people, and the death toll was 1.57 million [2]. Meanwhile, stroke also increased the risk of neurodegenerative diseases such as vascular dementia, Parkinson's disease, and Alzheimer's disease [3]. Ischemic stroke (IS) is the major type of stroke. It can damage sensory processing, communication, cognition, and movement of patients, which not only brings great inconvenience and pressure to patients but also brings heavy burden to the society [4, 5]. At present, tissue plasminogen activator is the most widely used treatment. However, this therapy is limited due to narrow therapeutic time window and various health adverse effects [6]. Recently, TCM characterized by multiherbs and multitargets treatment strategies has shown its unique advantages, and some of them have also been proven to be effective in treatment of IS [7–10].

Mi-Jian-Chang-Pu formula (MJCPF) is a well-known “Hui minority” medicine, which was firstly recorded in Huihui Prescription in the Yuan Dynasty. It is composed of *Crocus sativus L*. and *Acorus tatarinowii Schott*. According to ancient records, it was traditionally used for strengthening the spleen and resolving phlegm, promoting blood circulation, and removing blood stasis. According to modern clinical and animal researches, it was proved to be effective in the treatment of stroke, evidenced by the improved hemiplegia and language dysfunction of patients caused by stroke, and the recovered neurological function after cerebral ischemia-reperfusion injury in rats [11, 12]. Meanwhile, the individual herbs of MJCPF, including *C. sativus* and *A. tatarinowii*, could also be used to treat IS. *β*-asarone, a main constituent of *A. tatarinowii*, could across blood-brain barrier and enter the central nervous system and then improve cognitive impairment [13, 14]. *C. sativus* could be used for treatment of variety of diseases, such as cerebral obstruction, cardiovascular diseases, inflammatory diseases, depression, learning, and memory disorders [15]. Safranal, crocin I, and crocin II are the main components of *C. sativus*, which could protect the ischemic reperfusion injury of stroke model rats by inhibiting the production of free radicals and improving the activity of antioxidant enzymes [16].

Although the main components or the individual herbs of MJCPF had been proven to possess neuroprotective activity previously, however, the underlying mechanisms are still unclear. More importantly, it is unknown whether the combination of *C. sativus* and *A. tatarinowii* plays a synergistic role in treatment of IS. Therefore, by using a rat model of middle cerebral artery occlusion (MCAO) to mimic IS, and by employing pharmacodynamics, pharmacokinetics, and metabolomics approaches, the neuroprotective mechanisms as well as the synergistic effects of MJCPF in the treatment of IS were investigated. Our research will provide new ideas and methods for the study of TCM formula.

## 2. Materials and Methods

### 2.1. Chemicals and Reagents

Hematoxylin (Beijing Zhongshan Jinqiao Biotechnology Co., Ltd.); paraformaldehyde (Shanghai Chemical Reagent Company of China Pharmaceutical Group); xylene (Kelon Chemical Reagent Factory); 2,3,5-triphenyltetrazolium chloride (TTC, Sigma-Aldrich Co., Ltd.); VEGF, caspase-3, GFAP, and NF-*κ*B antibodies (Abcam Co., Ltd. Cambridge, UK). Crocin I (CAS 42553-65-1, purity ≥98%, AF8061502), *β*-asarone (CAS 5273-86-9, purity ≥98%, AF7112004), isofraxidin (CAS 486-21-5, purity 98%), methyl jasmonate (CAS 71109-09-6, purity 95%), 3-methylindole (CAS 83-34-1, purity ≥99.3%, C20893000), butyl 4-hydroxybenzoate (CAS 94-26-8, purity ≥99.3%, C14228780), vedaprofen (CAS 71109-09-6, purity ≥99.9%, V105300), 1-myristoyl-2-hydroxy-sn-glycero-3-phosphocholine (CAS 326495-21-0, purity ≥99.8%, ZZSI18041705), salsolinol (CAS 27740-96-1, purity ≥98%, ESI1764), stearic acid (CAS 57-11-4, purity ≥98%, wkq17040604), butyl 4-hydroxybenzoate (CAS.94-26-8, purity ≥99.3%, C14228780), tryptophan (CAS 73-22-3, purity ≥98%, wkq16090705), pantothenic acid (CAS 137-08-6, purity ≥97.5%, BP0297-LVY), and astragaloside IV (CAS 84687-43-4, purity ≥98%, CB8323949) were purchased from Chengdu AIFA Biotechnology Co., Ltd. (Chengdu, China). LC grade methanol and acetonitrile (Thermo Fisher Technology Co., Ltd.) and all the other reagents were of analytical grade.

### 2.2. Sample Preparation

MJCPF sample was prepared according to our previous study [17]. Firstly, 100 g of dried stems of *A. tatarinowii* was extracted by employing steam distillation approach for 3 h. Then oil was collected, and the residue was reextracted with water (1000 ml, 2 × 3 h); the filtrates were combined, evaporated, and mixed with the oil to obtain *A. tatarinowii* (23 g) sample. Secondly, 100 g of *C. sativus* was smashed and sieved through a 200-mesh sieve to obtain *C. sativus* sample. Thirdly, 50 g of powdered *C. sativus* was added in 11.5 g of *A. tatarinowii* sample and mixed thoroughly to obtain MJCPF sample. For animal experiments, *A. tatarinowii*, *C. sativus*, MJCPF, *β*-asarone, and crocin I were suspended in 0.5% CMC-Na aqueous solution, respectively. Meanwhile, the contents of *β*-asarone and crocin I in MJCPF sample were determined by employing LC method, which had been reported by our previous study [17]. And, the results were mainly used to calculate the dosages of *β*-asarone and crocin I in animal experiment, to ensure the dosage of *β*-asarone was consistent with it in *A. tatarinowii*, and similarly, crocin I was consistent with it in *C. sativus*.

### 2.3. Animal Experiment and MCAO Model Establishment

Animal experiment was conducted in accordance with the laboratory animal principles and protocols under the guidelines of the Bioethics Committee of the Ningxia Medical University (license number: NXMU-2018092). 150 three-month-old male Sprague-Dawley rats (weighing 280 ± 20 g) were provided by the Experimental Animal Center of Ningxia Medical University (license number: SCXK (Ning)-2015-0001). All animals were acclimated for one week in a clean environment with constant temperature and humidity (12 h/12 h light/dark cycle at 26°C and relative humidity of 65% ± 5%) and were fed and given water ad lib.

For pharmacodynamics experiment, the rats were randomly divided into 9 groups with 12 rats in each: sham group (N, vehicle), model group (M, vehicle), positive group (P, 36 mg/kg/day nimodipine), crocin I group (A1, 40 mg/kg/day), *β*-asarone group (B1, 15 mg/kg/day), crocin I + *β*-asarone group (A1B1, 55 mg/kg/day), *C. sativus* group (A, 580 mg/kg/day), *A. tatarinowii* group (B, 480 mg/day), and MJCPF group (AB, 1060 mg/kg/day). 0.5% CMC-Na was used as vehicle with an equal volume (100 g/ml body-weight) for each rat. Treatments were administered once daily by oral gavage for 14 consecutive days. After the last administration, rats were fasted for 12 h, MCAO surgery was performed according to the reference by H Nagasawa and K Kogure. [18]. After 2 h of sustained MCAO, reperfusion was conducted, and the rats in sham operation group received the same surgical procedures except that the occluding monofilament was not inserted.

For pharmacokinetic test, the rats were randomly divided into 7 groups with 6 rats in each: blank group, crocin I group (A1, 40 mg/kg/day), *β*-asarone group (B1, 15 mg/kg/day), crocin I + *β*-asarone group (A1B1, 55 mg/kg/day), *C. sativus* group (A, 580 mg/kg/day), *A. tatarinowii* group (B, 480 mg/day), and MJCPF group (AB, 1060 mg/kg/day). Serial blood samples were taken at 0, 0.083, 0.167, 0.25, 0.5, 0.75, 1, 2, 4, 6, 8, 10, 12, and 24 h after orally administration, placed in centrifuge tubes containing heparin sodium, centrifuged at 2000 rpm for 20 min, and stored at -80°C until analysis.

### 2.4. Pharmacodynamics Evaluation

#### 2.4.1. Neurological Deficit Evaluation

24 h after reperfusion, all rats were tested for sensorimotor ability according to the Longa's five-point scale (0, no neurologic deficit; 1, a slight forelimb flexion; 2 severe forelimb flexion and decreased resistance to lateral push; 3, spontaneous rotation or contralateral rotation to the affected side; 4, consciousness reduced and unable or difficult to move; 5, no response to stimulation).

#### 2.4.2. Behavior Evaluation

*(1) Forearm Grip Strength Test*. Each rat was held horizontally by the tail and allowed to grasp a grid which connected to a high-precision force meter and gently dragged backward until the grip was released, then the forearm grip strength was automatically recorded by the meter.

*(2) Balance Beam Walking Test*. The rats were trained to cross a standard balance beam (120 cm in length, 2 cm in width, 1 cm in thickness, and 80 cm off the floor) 3 times a day for 7 days before operative procedure. One end of the beam was connected to a cassette (20 cm × 20 cm × 20 cm) to stimulate rat to pass through the beam with appropriate noise. The degree of motor impairment was assessed using 6-point evaluation system below: 0, falls directly; 1, can lie on the beam but cannot move; 2, tries to pass the bar but falls off; 3, slides exceed 50% of the total number of steps; 4, slides more than one time but less than 50% of the total number of steps; 5, passed but injured hind limb slipped one time; 6, passed smoothly.

#### 2.4.3. Infarct Volume Measurement

The infarct volume was measured by applying TTC staining method. Rats were decapitated after the above behavioral experiments, the blood samples were obtained from the femoral artery, the brain tissue was removed immediately and cut into 5 coronal slices averagely, then immersed in 1.5% TTC for 45 min at 37°C in the dark, and turned them every 15 min. The results were analyzed by measuring the normal (red) and infarct volume ration of brain tissue (white).

#### 2.4.4. Histopathological and Immunohistochemical Staining (IHC) Tests

For brain histology, the tissue was fixed in 4% paraformaldehyde for 24 h, then the brain slices were rinsed, dehydrate, paraffin embedded, sectioned, and stained with hematoxylin-eosin (HE). The histopathological changes were observed under optical microscope.

For IHC, the brain slices were dehydrated, paraffin-embedded, and sectioned, then the levels of VEGF, GFAP, caspase-3, and NF-*κ*B were determined by using microscope and image analysis software.

### 2.5. Metabolisms Analysis

#### 2.5.1. Sample Preparation for Metabolisms Analysis

*(1) Preparation of Serum and Brain Sample Solutions*. Serum sample, collected from rats of pharmacodynamics experiments, was extracted with acetonitrile at a ratio of 1 : 5, vortexed for 60 s, and centrifuged at 13000 rpm at 4°C for 10 min. The supernatant was used for UHPLC-QTOF-MS/MS analysis. The brain tissue was accurately weighed and homogenized in water (mass ratio 1 : 1), then 100 *μ*l suspension was spiked with 100 *μ*l methanol acetonitrile (volume ratio: 1 : 1) and vortexed for 60 s, maintained at 4°C for 10 min, and then centrifuged at 13000 rpm at 4°C for 10 min, and the supernatant was taken for UHPLC-QTOF-MS/MS analysis.

*(2) Preparation of Quality Control Samples*. Quality control (QC) samples of serum and brain were, respectively, prepared by mixing 10 *μ*l aliquots from each sample and added with internal standard solution (seen in Supplementary File S1). QC sample was injected at regular intervals (every 10 samples) throughout the analytical run to monitor the stability of the entire analysis process.

#### 2.5.2. UHPLC-QTOF-MS/MS Conditions

UHPLC analysis was performed on an Agilent 1290 Infinity II system. A ZORBAX RRHD Eclipse Plus C_18_ column (50 mm × 2.1 mm, 1.8 *μ*m) with a binary solvent system (A-0.1% formic acid-water and B0.1% formic acid-acetonitrile) was utilized. The gradient elution was 1%-20% B at 0-1 min, 20%-50% B at 1-5 min, 50%-70% B at 5-10 min, 70%-85% B at 10-15 min, 85%-99% B at 15-16 min, 99% B at 16-17.5 min, 99%-1% B at 17.5-18.5 min, and 1% B at 18.5-20 min. The column temperature was 40°C, and injection volume was 1.5 *μ*l.

QTOF-MS/MS analysis was conducted on Agilent quadrupole time-of-flight 6545 mass spectrometer equipped with an electrospray dual spray ion source (Dual AJS ESI). The parameter settings were as follows: gas temperature, 350°C; capillary voltage, 3500 V; drying gas temperature, 350°C; drying gas flow, 12 l/min; nebulizer pressure, 40 psi; sheath gas temperature, 350°C; sheath gas flow, 10 l/min; fragmentor, 130 V; skimmer, 65 V; mass scanning range, *m*/*z* 100-1000.

#### 2.5.3. Method Validation

The developed UHPLC-QTOF-MS/MS analysis method was validated, mainly including extraction recovery, precision, matrix effects, and stability to ensure the reliability and stability of the analysis method. The detailed processes were showed in Supplementary File S1.

#### 2.5.4. Data Processing and Multivariate Analysis

The original datasets of all samples were firstly converted into mz.data using Agilent MassHunter Qualitative software, and then imported to R software package for peak identification, base-line correction, and normalization; a three-dimensional matrix consisting of sample name, retention time, peak intensities, *m*/*z* value was obtained. SIMCA (version 14.1, Umetrics AB, Umea, Sweden) was adopted for multivariate statistical analysis of the normalized data matrices, principal component analysis (PCA), and partial least squares discriminant ananlysis (PLS-DA). PLS-DA modeling analysis was implemented for the first principal component, and the model quality was verified by 7-fold cross validation. Variable importance in the projection (VIP) value was obtained from the PLS-DA model. Following, the permutation test was employed to change the sequence of classified variable *Y* randomly and repeatedly (200 times) to obtain different random *Q*^2^ values, so as to further evaluate the effectiveness of the model. Univariate analysis *t*-test was performed by employing SPSS (18.0). Metabolite peaks with VIP value greater than 1 and *p* value less than 0.05 were considered as significative differential metabolites. The relevant metabolic pathways in serum and brain sample were analyzed and identified by online database, including HMDB, KEGG, and Metabo Analyst.

### 2.6. Pharmacokinetic Evaluation

#### 2.6.1. Sample Preparation for Pharmacokinetic Analysis

*(1) Preparation of Plasma and Brain Sample Solutions*. Plasma sample, obtained from rats of pharmacokinetic test, was extracted with acetonitrile (containing 10 *μ*g/ml of matrine) at a ratio of 1 : 5, vortexed for 60 s, and centrifuged at 13000 rpm at 4°C for 10 min. The supernatant was used for LC-MRM-MS/MS analysis.

*(2) Preparation of Quality Control Samples*. 100 *μ*l of blank plasma (plasma obtained from rats of blank group) was added with 500 *μ*l of acetonitrile containing internal standard solution (seen in Supplementary File S2) to precipitate protein. After centrifuged at 13000 rpm 4°C for 10 min, 100 *μ*l of supernatant was added with 100 *μ*l of water, and then 100 *μ*l of standard solution with 8.76 *μ*g/ml of safranin I and 8.04 *μ*g/ml *β*-asarone was added as QC samples.

#### 2.6.2. LC-MRM-MS/MS Conditions

HPLC analysis was performed on Thermo Accela AS system by using a Symmetry® C18 (100 mm × 2.1 mm, i.d., 3.5 *μ*m) column with a binary solvent system (water containing 0.1% formic acid as mobile phase A and acetonitrile as mobile phase B). The gradient elution set as follows: 80% A from 0-1 min, 80-10% A from 1-3 min, 10% A from 3-4 min, 10-80% A from 4-4.1 min, and 80% A from 4.1-6 min. The flow rate was 0.3 ml/min with the temperature maintained at 25°C, and the injection volume was 10 *μ*l.

MRM-MS/MS analysis was performed on a Thermo TSQ Quantum Access MAX system equipped with an electrospray ionization (ESI) source. The spray voltages in the positive and negative ion modes were 4.0 kV and 2.5 kV, respectively; capillary temperature, 320°C; vaporizer temperature, 350°C; sheath gas pressure, 35 psi; aux gas pressure, 10 psi.

#### 2.6.3. Method Validation

The developed LC-MRM-MS/MS analysis method was validated, mainly including linear, repeatability, extraction recovery, precision, matrix effects, and stability to ensure the reliability and stability of the analysis method. The detailed processes were showed in Supplementary File S2.

#### 2.6.4. Data Analysis

DAS3.0 was used to calculate the *in vivo* pharmacokinetic parameters by a noncompartment model. The peak area of plasma sample was obtained employing Xcalibur 2.2 data processing system, and the concentrations of crocin I and *β*-asarone in plasma were calculated using linear regression equations. Time-concentration-time curves were plotted applying Prism 8.0 (GraphPad Software Inc., San Diego, CA).

### 2.7. Statistical Analysis

All of the experimental results were described as mean ± SD and were analyzed by using SPSS software (version 18.0, IBM SPSS Statistics, IBM Corp., Armonk, New York, NY, USA). Data were performed by one-way analysis of variance ANOVA or *t*-test, and the *p* value less than 0.05 was considered statistically significant.

## 3. Results

### 3.1. Effects on Neurological Deficit, Behavioral, and Morphological Characterization

#### 3.1.1. Effects on Body Weight Change

**Figure 1:**
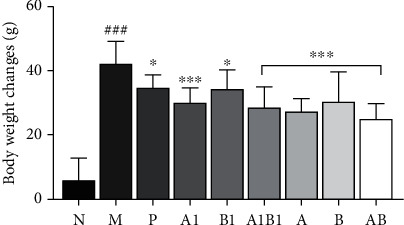
Body weight changes of rats in sham group, model group, and different administration groups. Drug treatment can reduce the weight change of MCAO rats. ^∗^*p* < 0.05, ^∗∗∗^*p* < 0.001, as compared to M group; ^###^*p* < 0.001, as compared to N group.

The body weight before operation and after drug intervention was recorded, respectively, to estimate the effect of MJCPF on MCAO rats. As shown in Figure 1, the body weight change of rats in MCAO model group was significantly enlarged as compared to the sham rats (*p* < 0.001), specifically, a rapid weight loss was observed in rat after cerebral ischemia reperfusion injury. After drug intervention, the body weight change was significantly reduced (*p* < 0.05) as compared with the model group, implied that no matter the single compound, individual herb, or their combinations, the rapid weight loss caused by cerebral ischemia was improved. Meanwhile, A1B1 and AB combinations exhibited slightly stronger effects than the single compound and individual herb alone.

#### 3.1.2. Effects on Neurological Deficit

**Figure 2:**
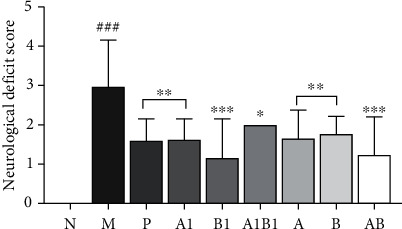
The neurological deficit scores of rats were evaluated after brain ischemia. Drug treatment can decrease obviously the neurological function scores. ^∗^*p* < 0.05, ^∗∗^*p* < 0.01, and ^∗∗∗^*p* < 0.001, as compared to M group; ^###^*p* < 0.001, as compared to N group.

After 24 h of reperfusion, rats in each group were subjected to neurological deficit score test, and the results were showed in Figure 2. The neurological deficit score of MCAO model rats was significantly higher than that of sham-operated rats (*p* < 0.001). After treatment with drugs, the neurological function scores were all obviously decreased (*p* < 0.05) when compared with MCAO model group. Abnormally, both A1 and B1 showed significant effects on neurological deficit score of rats, whereas their combination A1B1 presented weaker effects than A1 or B1. Interestingly, AB combination exhibited stronger effects than the individual herb A and B, which implied a synergistic effect of AB.

#### 3.1.3. Effects on Behavioral Characterization

**Figure 3:**
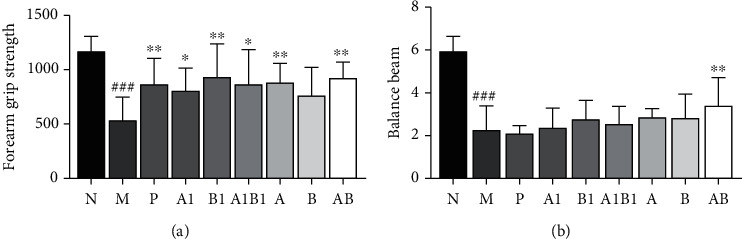
The behavioral tests of rats in each group were evaluated. Drug treatment can meliorate the behavioral scores. (a) Forearm grip strength of rats. (b) Balance beam test of rats. ^∗^*p* < 0.05, ^∗∗^*p* < 0.01, as compared to M group; ^###^*p* < 0.001, as compared to N group.

As showed in Figure 3, in grip strength test, a significantly neurological impairment was observed in MCAO model rats in which the forearm grip strengths of MCAO model rats were significantly decreased as compared with the sham rats (*p* < 0.001). After treatment with the single compound, individual herb, or their combinations, the strengths of MCAO rats were all significantly enhanced as compared with the rats of model group (*p* < 0.05). Thoroughly different from the grip strength test, in balance beam experiment, the scores of rats in MCAO model group were significantly higher than the sham rats. Although treated with different drugs, the scores of MCAO rats were not obviously decreased except AB combination, implying that AB combination exhibited stronger neuroprotective activity than the single compound, individual herb, or A1B1 combination.

#### 3.1.4. Effects on Infarcted Brain by Using TTC Staining

**Figure 4:**
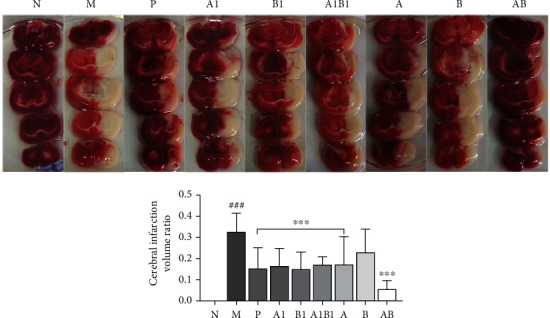
TTC staining results of brain tissue. Five brain coronal sections were selected for tetrazolium chloride (TTC) staining. Red stain represents normal tissues; white represents the infarct region. ^∗∗∗^*p* < 0.001, as compared to M group; ^###^*p* < 0.001, as compared to N group.

TTC staining results were showed in Figure 4, which a red color indicated normal brain tissue, and a pale grey color indicated infarcted tissue. An obviously ischemic lesions were observed in rats of MCAO model group (*p* < 0.001), whereas no infarcted area was monitored in sham rats, implied the reliable of our IS model induced by MCAO surgery. After intervention with drugs, whether the single compound, individual herb, or their combinations, a significantly declined cerebral infraction volume ratio was observed (*p* < 0.01). In addition, AB combination exhibited better neuroprotective effects than A1, B1, A, B, and A1B1. The TTC results also implied that the combination of A and B exert synergistic effect.

#### 3.1.5. Effects on Histopathological Changes by Using He Staining

**Figure 5:**
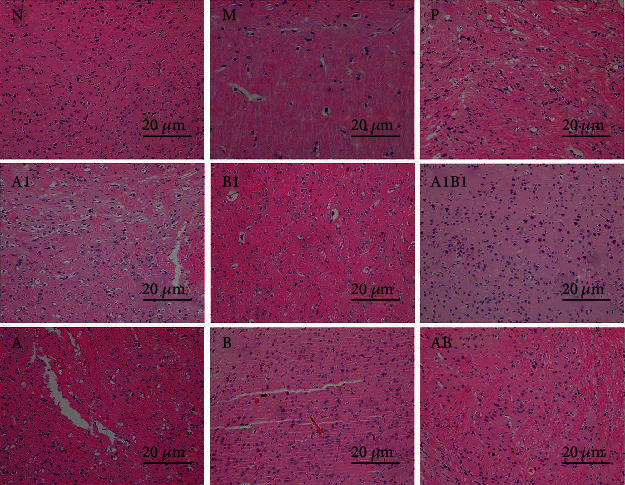
Histopathological examination of brain tissues by HE staining. The histopathological changes of the treatment group were meliorated in different degrees.

Histopathological changes in the brain tissue of rats were presented in Figure 5, in which an apparent damaged neuron structure, swelling of neurons, nuclear condensation, neuronal loss, and numerous vacuolated spaces were found in rats of MCAO model group. However, these pathological changes were alleviated to varying degrees in the treatment, especially in the combination group of AB, which were similar to that of the sham group. The results further discovered that the combination of AB, also named MJCPF, seemed to act synergistically.

#### 3.1.6. Effects on Histopathological Changes by Using IHC

**Figure 6:**
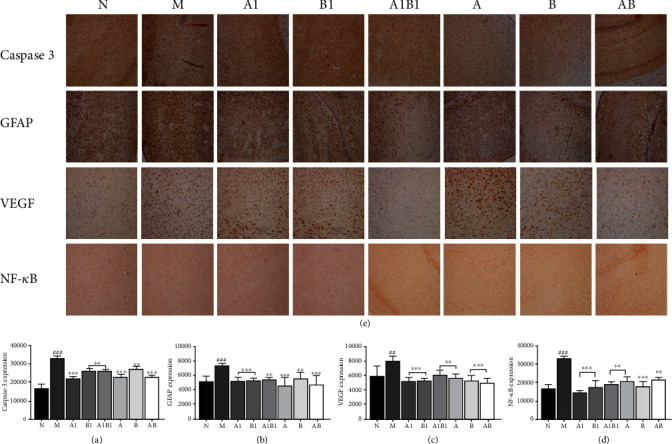
Effect of drug on protein levels of caspase3, GFAP, VEGF, and NF-*κ*B in cortex of MCAO rats. Drug treatment significantly reversed protein expression. F: Brain coronal sections were selected for ICH staining; (a–d) were the statistical results of protein expression of caspase 3, GFAP, VEGF, and NF-*κ*B, respectively. ^∗∗^*p* < 0.01, ^∗∗∗^*p* < 0.001, as compared to M group; ^##^*p* < 0.01, ^###^*p* < 0.001, as compared to N group.

As illustrated in Figure 6, when compared with the sham group, the expressions of GFAP, VEGF, caspase-3, and NF-*κ*B in brain tissue of rats of MCAO model group were all increased significantly (*p* < 0.001). All treatment groups could significantly reverse the above abnormal changes, implying that, MJCPF and its single compounds as well as individual herbs could inhibit neuro-inflammation in MCAO rats.

### 3.2. Metabolomics Analysis

#### 3.2.1. Methodological Validation

By applying serum and brain QC samples, the stability of the entire analysis process was monitored, and the condition of UHPLC-QTOF-MS/MS was optimized. Data acquisition in both positive and negative ion modes indicated a good repeatability, stability, precision, matrix effect, and extraction recovery of our method (Supplementary File S1).

#### 3.2.2. Data Analysis with Multivariate Statistical Analysis

**Figure 7:**
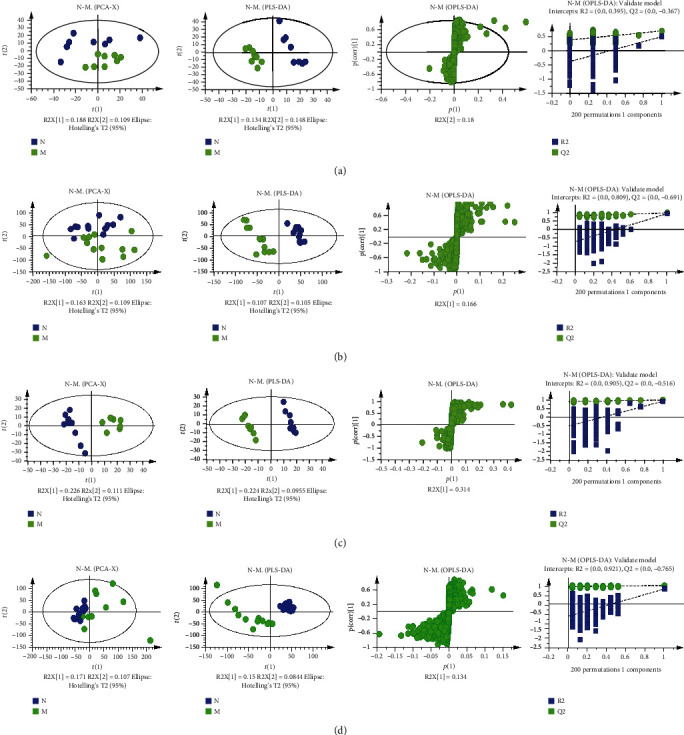
Multivariate statistical analysis of the sham and MCAO model groups for serum/brain samples in positive and negative ion modes. (a) PCA, PLS-DA, scatter plot, and permutation test of serum sample in positive ion mode. (b) Analysis of serum sample in negative ion mode. (c) Analysis of brain sample in positive ion mode. (d) Analysis of brain sample in negative ion mode.

Both PCA and PLS-DA score plots were employed to observe the distinct separation between the sham and MCAO model groups. As shown in Figures 7(a)–7(d), except the PCA score plots, there was an obvious separation between the two groups in PLS-DA score plots, indicating that metabolic profiles of serum and brain tissue in MCAO rats were significantly changed after a cerebral ischemia reperfusion injury. S-plots were used to identify altered metabolites which were located in the upper right or lower left quadrant and farther away from the origin. Moreover, the results of permutation tests with 200 iterations showed that the OPLS-DA model were stable and well-fitted, with good predictive ability and reliability.

#### 3.2.3. Differential Metabolites Identification and Metabolic Pathway Analysis

**Figure 8:**
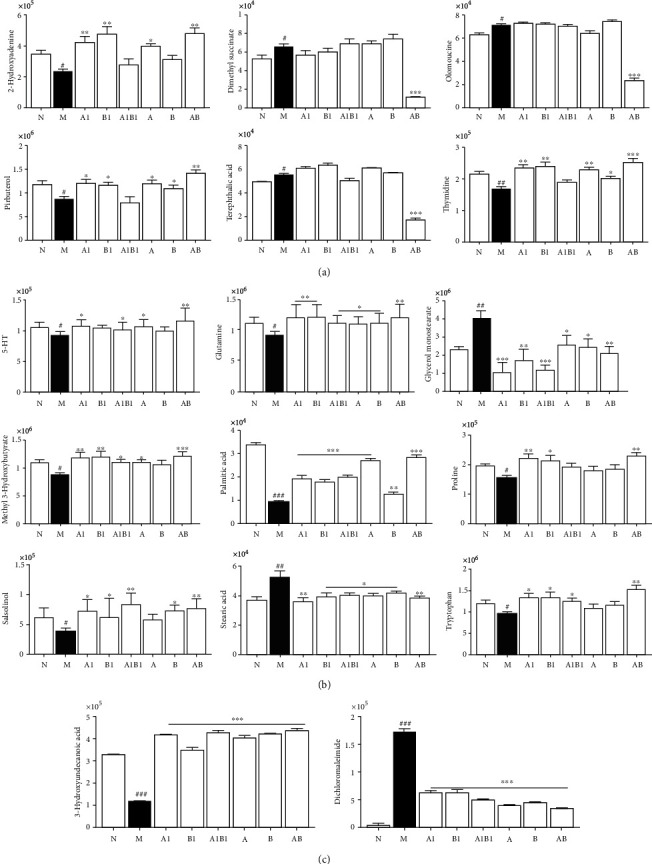
Synergistic effects of MJCPF on differential metabolites. ^∗^*p* < 0.05, ^∗∗^*p* < 0.001, and ^∗∗∗^*p* < 0.001, as compared to M group; ^#^*p* < 0.05,^##^*p* < 0.01, and ^###^*p* < 0.001, as compared to N group. (a) Differential metabolites identified in brain sample in positive ion mode. (b) Serum sample in positive ion mode. (c) Serum sample in negative ion mode.

**Figure 9:**
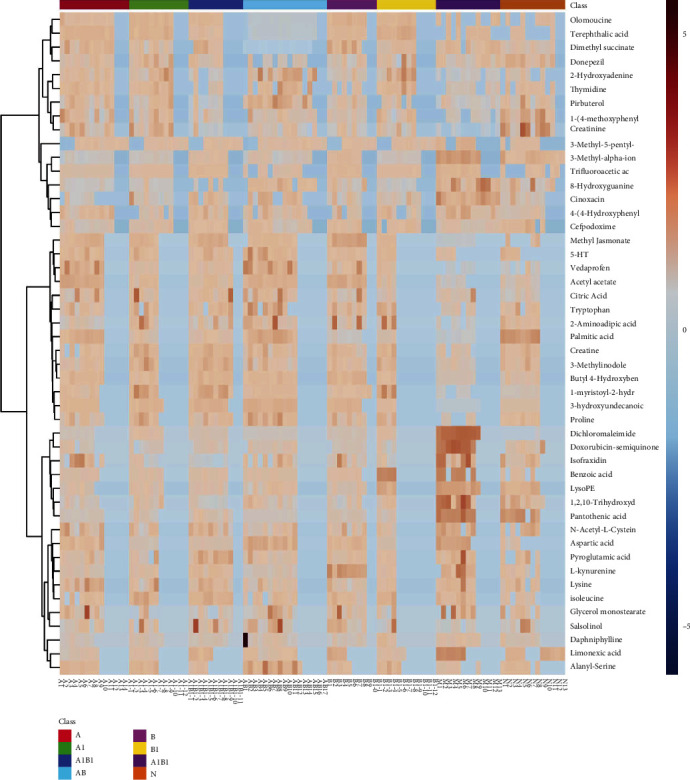
Heat map of Spearman correlation analysis between metabolites in both serum and brain of each group. The abscissa represents different experimental groups, and the ordinate represents the different metabolites in this group, and the color blocks in different positions represent the relative expression levels of metabolites in corresponding positions. Orange-red indicates high expression of this substance, while blue indicates low expression of this substance.

Based on the OPLS-DA model and S-plots, the differential metabolites in both serum and brain samples between sham and MCAO model groups were elucidated. On the basis of *p* < 0.05 and *VIP* > 1, 35 differential metabolites in serum samples (Table 1) and 18 metabolites in brain samples (Table 2) were identified, respectively. Interestingly, all of the differential metabolites in MCAO rats were restored to varying degrees in the treatment, seen in Figure 8 and Supplementary File S3, especially in the combination group of AB, 17 of which were more significantly restored than the other treated groups, as presented in Figure 8. Thus, the results of metabolomics further implied that the combination of A and B exert synergistic effect. Meanwhile, the cluster analysis of metabolites was showed as a heat map in Figure 9.

**Figure 10:**
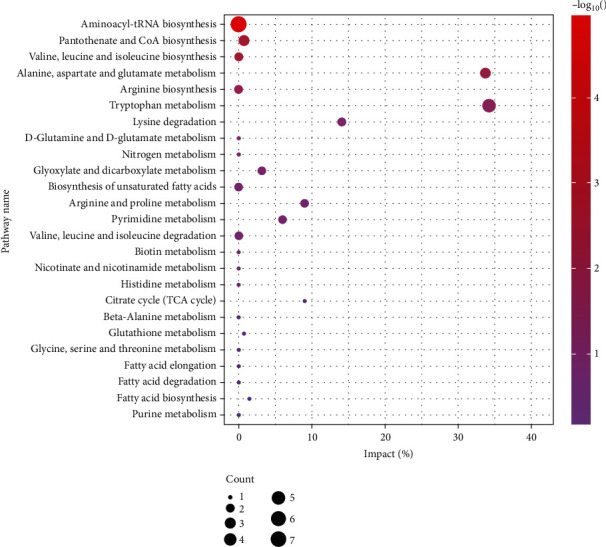
Bubble plot of differential metabolites' pathway enrichment between sham and MCAO model groups. Each bubble in the bubble chart represents a metabolic pathway. The abscissa represents impact, and the ordinate represents metabolic pathways. The size of the dot indicates the number of different metabolites, and the color of the dot indicates the *p* value.

In order to find the underlying relevance between the differential metabolites involved in IS and the drug intervention, the main biological metabolic pathway analysis was performed using Metabo Analyst 5.0 online database based on the identified differential metabolites in serum and brain samples. The results were shown in Figure 10, in which the identified metabolites were associated with 7 main metabolisms, namely, aminoacyl-tRNA biosynthesis, pantothenate and CoA biosynthesis, valine, leucine and isoleucine biosynthesis, alanine, aspartate and glutamate metabolism, arginine biosynthesis, tryptophan metabolism, and lysine degradation.

#### 3.2.4. Beneficial and Synergistic Effect of MJCPF on Metabolites

In order to explore whether the combinations of individual herbs or the major active components of MJCPF possess synergistic neuroprotective effects against the differential metabolites, both PCA and PLS-DA models were employed to estimate the overall regulation trends in different treated groups.

**Figure 11:**
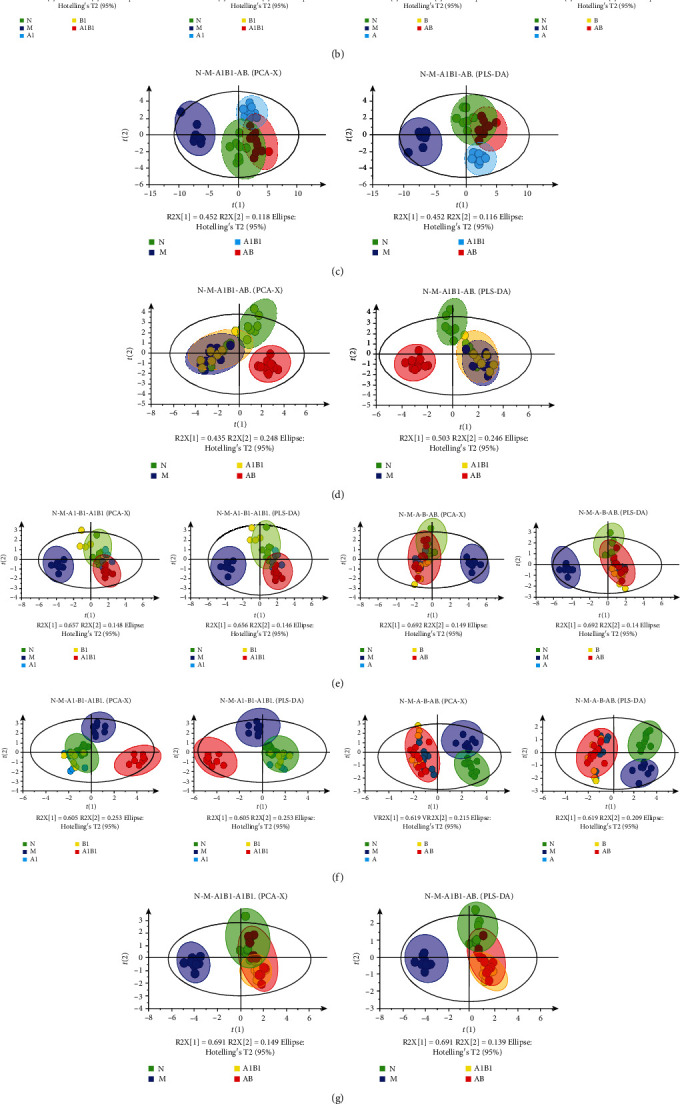
PCA and PLS-DA analysis of differential metabolites in serum and brain samples. (a, e) PCA and PLS-DA analysis of serum sample of different groups in positive and negative ion modes, respectively. (b, f) PCA and PLS-DA analysis of brain sample of different groups in positive and negative ion modes, respectively; (c, g) PCA and PLS-DA analysis of serum sample of N, M, A1B1, and AB groups in positive and negative ion modes, respectively; (d, h) PCA and PLS-DA analysis of brain sample of N, M, A1B1, and AB groups in positive and negative ion modes, respectively.

Concerning the positive ion mode, as shown in Figure 11(a), there were obvious separation trends between the sham (N) and MCAO model (M) groups in both PCA and PLS-DA analysis of serum sample. Meanwhile, in PCA model of serum sample, PCA and PLS-DA models of brain sample, all of the treatment groups including A1, B1, A, B, A1B1, and AB, were partly overlapped with the sham group. The above results indicated the beneficial effects of all drugs on MCAO rats. Totally different from the serum sample (Figure 11(b)), a slight overlapped phenomenon was observed between the sham and MCAO model groups in brain sample. Interestingly, as shown in Figure 11(b), although both A1 and B1 achieved good separations from the MCAO model group, their combination A1B1 totally overlapped with the MCAO model group. Meanwhile, the individual herbs A and B overlapped with the sham group, and partly overlapped with the MCAO model group, but their combination AB showed an obvious separation trend from both sham and MCAO model groups. The above results revealed that the beneficial effects of A, B, and AB were stronger than A1, B1, and A1B1. Furthermore, in serum PCA and PLS-DA analysis, as shown in Figure 11(c), the combination of AB was closer to sham than A1B1; meanwhile, in brain PCA and PLS-DA analysis, seen in Figure 11(d), the combination of A1B1 totally overlapped with the MCAO model group, whereas AB combination was close to the sham group and distinctly away from the MCAO model group.

Concerning the negative ion mode, as shown in Figure 11(e), all of the intervention groups partly overlapped with the sham group. Interestingly, the combination of A1B1 showed a different effect from the single compounds A1 and B1, whereas the AB combination showed a similar effect to its individual herbs A and B (Figure 11(f)). In addition, in serum sample (Figure 11(g)), both the A1B1 and AB showed the similar effects which partly overlapped with sham group; whilst and in brain sample (Figure 11(h)), A1B1 and AB also showed same effects which overlapped with each other.

All in all, by integrating serum and brain samples, positive and negative ion modes, and PCA and PLS-DA analysis, the results of the metabolism showed that there indeed existed a synergistic effect of MJCPF on differential metabolites.

### 3.3. Pharmacokinetic Evaluation

#### 3.3.1. Methodological Validation

The regression equations of crocin I and *β*-asarone were calculated by linear least squares regression, and the results of precision, reproducibility, matrix effect, extraction recovery, and stability complied with the requirements. All of the data were showed in Supplementary File S2, which indicated that this method was reliable and accurate.

#### 3.3.2. Beneficial and Synergistic Effect of MJCPF on Pharmacokinetic Parameters

**Figure 12:**
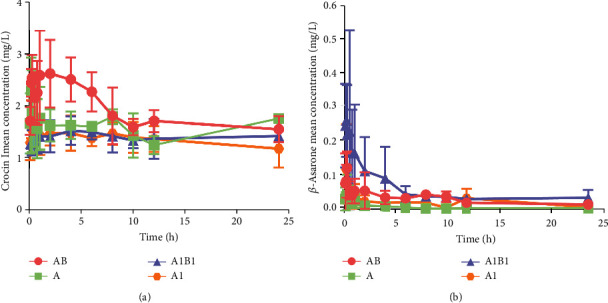
Serum concentration-time curves of different treated groups. (a) Serum concentration-time curve of crocin I. (b) Serum concentration-time curve of *β*-asarone. The abscissa represents time (h), and the ordinate represents serum drug concentration.

The concentration-time curves and pharmacokinetic parameters of A1 (crocin I) and B1 (*β*-asarone) were presented in Figure 12, Tables 3 and 4, respectively. Concerning crocin I, the value of C_max_, T_max_, and AUC _(0-t)_ in A1B1, A, and AB groups were increased as compared with A1 group, especially AB group which most of the parameters were higher than the others. The results revealed that A1B1, AB, and A could promote the absorption of crocin I. Concerning *β*-asarone, the combinations of A1B1 and AB increased the value of C_max_, AUC _(0-t)_ and AUC _(0-_∞_)_ of *β*-asarone, and A1B1 decreased V_Z/F_ and CL_Z/F_, which implied the combination of crocin I and *β*-asarone together could promote the absorption and improve the clearance and allow the *β*-asarone in the effective concentration range. However, an obviously decreased *C*_max_, *T*_max_, AUC_(0 − *t*)__,_ and *AUC* (0 − ∞), as well as increased V_Z/F_ and CL_Z/F_ were observed in B group as compared with B1, implied the components contained in *A. tatarinowii* might influence the absorption of *β*-asarone. The difference of pharmacokinetic parameters verified that the combination of AB and A1B1 promoted the absorption of crocin I and *β*-asarone, respectively.

## 4. Discussions

Stroke, one of the main causes of human death, has high morbidity, mortality, and recurrence rates [19]. Due to its complex pathogenesis, there are no satisfactory treatments available currently. MJCPF is an important minority medicine of TCM, which had been used in the clinical treatment of stroke. On the other side, it is also a simple TCM formula which composes only two herbs, including *C. sativus* and *A. tatarinowii*. Beside describing the underlying neuroprotective mechanisms, we also want to know the respective roles of these two individual herbs in the treatment of IS, specifically, whether the combination of *C. sativus* and *A. tatarinowii*, even the combination of the major active compounds of crocin I and *β*-asarone, exert a synergistic neuroprotective effect. To advance this study, by integrating pharmacodynamics, metabolomics, and pharmacokinetics, we herein reveal as follows: (1) the combination indeed exerted synergistic effects against IS; (2) and more importantly, we found that the combination of *C. sativus* and *A. tatarinowii,* other than crocin I and *β*-asarone, exhibited a remarkable synergistic effect.

It is well known that cerebral infarction volume is an important factor for evaluation of cerebral ischemia injury. It can also be used to access the benefit of potential therapies on stroke outcome. Our results showed that, no matter the single main active compounds, including crocin I (A1) and *β*-asarone (B1), the individual herbs, including *C. sativus* (A) and *A. tatarinowii* (B), or their respective combinations A1B1 and AB (MJCPF), all of the them could reduce the infarction volume significantly. And more importantly, the intervention effect of MJCPF was stronger than the others, in which the rats showed the smallest infarct volume. Besides infarction volume, the neurological deficit scores and body weight changes of MCAO rats were also evaluated, all of the results proved the protective efficacy of MJCPF on IS. The results initially implied that AB combination exerted a synergistic effect.

Furthermore, to observe whether it can promote the survival of neurons and play a key role in brain tissue repair, histopathological and IHC were determined. HE staining results revealed that all of our drugs, including A1, B1, A, B, A1B1, and AB had protective effect on brain injury by reducing the occurrence of neuronal loss, vacuolated spaces, and shrunken and disordered neurons. Meanwhile, we found that the recovery degree of the AB group was better than the other groups and was closer to the sham operation group, which further explained the synergistic effect of MJCPF. Futhermore, there were no obvious side effects on each organ or mortality were observed, suggesting the safety of single compounds, individual herbs, and their respective combinations.

IS often leads to elevated GFAP, VEGF, caspase-3, and NF-*κ*B levels in MCAO rats, which had been proved by published papers and our previously study [20–22]. Hypoxia-inducible angiogenic peptide VEGF was believed to have played a key role in chronic neuroinflammation by interacting with inflammatory cells. GFAP, an intermediate filament cytoskeletal protein, was considered an important parameter of glial reactivity. In particular, the increase in GFAP was often used to examine glial cell reactivity after neural injury. Caspase-3 played a key role in apoptosis and could modulate the regenerative response after stroke [23]. NF-*κ*B is an important transcriptional regulator which could aggravate cerebral ischemia/reperfusion injury by promoting inflammation, inducing apoptosis, and free radical injury [24], and it had been proved that stroke could lead to increased expression of NF-*κ*B [25]. Accordingly, the results of our immunofluorescent staining test were consistent with those reported in the literature and supported the above-mentioned conclusion. Specifically, the number of GFAP, VEGF, caspase-3, and NF-*κ*B positive cells, stained yellowish-brown, was increased obviously in the brain tissue of MCAO model rats as compared with the sham rats. However, after the intervention, the expressions of the above abnormal factors were all significantly decreased.

Currently, metabolomic is increasingly being used to understand the etiology and pathogenesis of IS. In the present study, UHPLC-QTOF-MS/MS-based metabolomics was carried out to estimate the potential synergistic neuroprotective effect and the possible mechanisms of MJCPF on MCAO rats by employing both the serum and brain metabolite profiling. A total of 53 differential metabolites were identified between MCAO and sham groups, and 18 of which were discovered from the brain tissue, the other 30 were from the serum sample. Interestingly, all the metabolites were reversed after drug intervention, and MJCPF had a significantly better reversal effect on 17 metabolites than individual herb intervention.

Among these metabolite, 7 endogenous metabolites were found to be directly involved in the metabolic pathway of amino acids, including aspartic acid, valine, N-acetyl cysteine, isoleucine, glutamine, lysine, and proline. Therefore, once cerebral ischemia occurred, amino acid metabolism was disordered [17]. Nucleoside metabolism, including purine and pyrimidine metabolisms, was identified as playing an important role in maintaining the brain's energy balance. 2-Hydroxyadenine is a hydrogenated derivative of guanine, and both 2-hydroxyadenine and 8-hydroxyguanine are the components of nucleic acids and are involved in the synthesis of genetic material [26]. In this study, the expression levels of 2-hydroxyadenine, thymine, and 8-hydroxyguanine were downregulated after brain injury, suggesting that nucleotide metabolism balance was related to the occurrence of cerebral ischemia. In addition, the tricarboxylic acid cycle (TCA) is a common metabolic pathway for carbohydrate, fat, and protein metabolism, and 2-hydroxyadenine served as fuel for the TCA, which suggested that the occurrence of IS was related to TCA [27]. Studies have shown that after brain injury, the expressions of citric acid, fumaric acid, and malic acid were decreased, indicating that the TCA pathway was inhibited [28]. The increased ketone body metabolism could provide alternative energy sources and to maintain free radical homeostasis during ischemia reperfusion injury [29]. 1-(4-methoxyphenyl)-4-methylpenta-1,4-pentadien-3-one, the precursor of ketone body, was significantly downregulated in MCAO model group in this experiment, which implied the ketone body metabolism was decreased. And consequently, an insufficient energy supply for brain tissue was occurred. Therefore, both our results and the published data proved that the ketone body metabolism was involved in the occurrence of IS [30]. Glutamine was proved possessing anti-inflammatory and antioxidant effects [31]. In this experiment, glutamine was significantly downregulated in MCAO rats, implied that glutamate metabolism was related to the occurrence of IS, which was also consistent with previous studies [32].

MJCPF is compose of *C. sativus* and *A. tatarinowii*, whose therapeutic efficacy is exerted by the combined actions of a mixture of constituents [33]. In order to reveal the different beneficial effects of the main bioactive compounds with or without other compounds and herbs, the pharmacokinetics experiment was performed, which could link the chemistry of MJCPF with its pharmacodynamics activity, and also could disclose and explain the synergistic effect. In this study, a rapid, accurate, and sensitive LC-MRM-MS/MS method was established for the pharmacokinetics analysis of crocin I and *β*-asarone in different groups, respectively. The results revealed that, no matter the combination of crocin I and *β*-asarone, or the combination of *C. sativus* and *A. tatarinowii*, all of which could promote the absorption of crocin I and *β*-asarone, respectively. The advantage of using TCM formula as a protective agent for treatment of IS was confirmed again from the perspective of pharmacokinetics.

## 5. Conclusion

In summary, the present study was the first to explore the synergistic neuroprotective effect of MJCPF on MCAO rat model of IS, as well as the possible mechanism by integration of pharmacodynamics, pharmacokinetics, and metabolomics. Our results demonstrated that the single compounds, the individual herbs, and their respective combinations could improve neurological functions, enhance motor coordination, reduce inflammatory responses, and restore all of 53 abnormal metabolites of MCAO rats. Interestingly, the neuroprotective effect of MJCPF was superior to that of other groups, which proving the synergistic therapeutic effect of MJCPF. Taken together, MJCPF exerted a stronger protective activity than the single compounds and individual herbs alone, and might be a potential candidate for IS prevention and treatment.

## Figures and Tables

**Table 1:** Differential metabolites identified in serum sample between sham and model groups.

No	Rt (min)	Exact mass	Formula	Identification	VIP	Trend	Ion mode
1	0.71	132.0808	C_9_H_9_N	3-Methylindole	1.69	↓	+
2	2.19	205.0972	C_11_H_12_N_2_O_2_	Tryptophan	1.28	↓	+
3	5.61	209.0847	C_10_H_12_N_2_O_3_	L-kynurenine	2.01	↑	+
4	0.68	164.0224	C_5_H_8_NO_3_S	N-acetyl cysteine	1.92	↑	+
5	2.14	134.0375	C_4_H_7_NO_4_	Aspartic acid	1.65	↓	+
6	0.70	147.0691	C_5_H_10_N_2_O_3_	Glutamine	2.02	↓	+
7	9.91	532.3409	C_27_H_52_NO_7_P	LysoPE (0 : 0/22 : 2(13z,16z)	1.74	↑	—
8	0.62	130.0425	C_5_H_7_NO_3_	Pyroglutamic acid	1.42	↑	+
9	16.43	381.1766	C_16_H_30_O_10_	1,2,10-Trihydroxydihydro-trans-linalyl oxide 7-O-beta-D-glucopyranoside	1.49	↑	—
10	2.13	147.1055	C_6_H_14_N_2_O_2_	Lysine	2.04	↑	+
11	0.66	193.0271	C_6_H_8_O_7_	Citric acid	1.22	↑	+
12	18.96	103.0316	C_4_H_6_O_3_	Acetyl acetate	1.64	↓	+
13	0.88	116.0633	C_5_H_9_NO_2_	Proline	1.72	↓	+
14	1.51	132.0946	C_6_H_13_NO_2_	Isoleucine	2	↑	+
15	18.94	118.0789	C_5_H_11_N0_2_	Valine	1.61	↓	+
16	7.91	162.0688	C_6_H_11_NO_4_	2-Aminoadipic acid	1.92	↓	+
17	17.64	163.9312	C_4_HCl_2_NO_2_	Dichloromaleimide	2.14	↑	—
18	3.31	195.1016	C_11_H_14_O_3_	Butyl 4-hydroxybenzoate	1.19	↓	+
19	1.51	132.0695	C_4_H_9_N_3_O_2_	Creatine	1.92	↓	+
20	13.77	257.2402	C_16_H_32_O_2_	Palmitic acid	2.12	↓	+
21	7.69	201.1553	C_11_H_22_O_3_	3-Hydroxyundecanoic acid	1.82	↓	—
22	1.47	180.1019	C_10_H_13_NO_2_	Salsolinol	1.29	↓	+
23	16.4	285.2788	C_18_H_36_O_2_	Stearic acid	1.64	↑	+
24	0.72	177.0949	C_10_H_12_N_2_O	5-HT	1.85	↓	+
25	10.34	526.3538	C_32_H_49_NO_5_	Daphniphylline	1.18	↑	—
26	3.31	223.0601	C_11_H_10_O_5_	Isofraxidin	1.58	↑	+
27	7.06	225.1485	C_13_H_20_O_3_	Methyl jasmonate	1.18	↓	+
28	7.32	468.3163	C_22_H_47_NO_7_P	1-Myristoyl-2hydroxy-sn-3-phosphocholine	2.14	↓	+
29	2.362	121.0295	C_7_H_6_O_2_	Benzoic acid	1.38	↑	—
30	11.01	377.1871	C_23_H_26_N_2_O_3_	4R-Hydroxysolifenacin	1.29	↓	—
31	18.27	220.1191	C_9_H_17_NO_5_	Pantothenic acid	1.33	↑	+
32	14.43	283.1693	C_19_H_22_O_2_	Vedaprofen	1.78	↓	+
33	2.13	119.0629	C_5_H_10_O_3_	Methyl 3-hydroxybutyrate	1.47	↓	+
34	2.37	359.3083	C_21_H_42_O_4_	Glycerol monostcarate	2.09	↑	+
35	18.91	546.1981	C_27_H_33_NO_11_	Doxorubicin-semiquinone	1.94	↑	—

**Table 2:** Differential metabolites identified in brain sample between sham and model groups.

No	Rt (min)	Exact mass	Formula	Identification	VIP	Trend	Ion mode
1	0.33	147.0652	C_6_H_10_O_4_	Dimethyl succinate	1.54	↑	+
2	0.72	114.0662	C_4_H_7_N_3_O	Creatinine	1.45	↓	+
3	18.75	501.1776	C_26_H_30_O_10_	Limonexic acid	1.69	↓	—
4	1.98	243.0975	C_10_H_14_N_2_O_5_	Thymidine	1.32	↓	+
5	0.73	168.0561	C_5_H_5_N_5_O_2_	8-Hydroxyguanine	1.16	↑	+
6	0.74	152.0567	C_5_H_5_N_5_O	2-Hydroxyadenine	1.31	↓	+
7	9.58	251.1653	C_15_H_24_O_3_	3-Methyl-5-pentyl-2-furanpentanoic acid	1.63	↑	—
8	3.56	177.0870	C_6_H_12_N_2_O_4_	Alanyl-serine	1.56	↓	+
9	17.74	167.0339	C_8_H_6_O_4_	Terephthalic acid	1.46	↑	+
10	10.40	203.1067	C_13_H_14_O_2_	1-(4-Methoxyphenyl)-4-Methyl-1,4-Pentadien-3-one	1.34	↓	+
11	16.53	380.2220	C_24_H_29_NO_3_	Donepezil	1.14	↓	+
12	0.74	426.0547	C_15_H_17_N_5_O_6_S_2_	Cefpodoxime	1.66	↑	—
13	0.65	261.0517	C_12_H_10_N_2_O5	Cinoxacin	1.54	↑	—
14	0.77	629.1512	C_30_H_30_O_15_	4-(4-Hydroxyphenyl)-2-butanone O-[2,6-digalloylglucoside]	1.16	↑	—
15	10.03	299.1615	C_15_H_18_N_6_O	Olomoucine	1.51	↑	+
16	1.51	241.1547	C_12_H_20_N_2_O_3_	Pirbuterol	1.19	↓	+
17	9.50	249.1860	C_16_H_26_O_2_	3-methyl-alpha-ionyl acetate	1.84	↑	—
18	4.37	112.9856	C_2_HF_3_O_2_	Trifluoroacetic acid	1.62	↑	—

**Table 3:** Pharmacokinetic parameters of crocin I after oral administration in mice of each group.

Parameters	A1	A1B1	A	AB
C_max_ (mg/l)	1.77 ± 0.35	1.91 ± 0.42	2.33 ± 0.66	3.24 ± 0.61
T_max_ (h)	0.18 ± 0.03	5.01 ± 9.41	0.36 ± 0.34	1.88 ± 2.42
t_1/2z_ (h)	25.63 ± 21.46	11.20 ± 4.78	9.29 ± 3.12	28.84 ± 17.96
AUC (0-t) (mg/l^∗^h)	20.49 ± 10.75	26.91 ± 8.95	21.89 ± 12.21	36.63 ± 13.56
AUC (0-∞) (mg/l^∗^h)	61.33 ± 52.62	34.91 ± 11.75	33.69 ± 12.06	86.80 ± 48.18
V_Z/F_ (l/kg)	20.28 ± 5.10	14.11 ± 5.64	209.33 ± 52.54	226.75 ± 67.22
CL_Z/F_ (l/h/kg)	1.11 ± 0.84	1.27 ± 0.49	20.23 ± 10.32	8.81 ± 5.12

**Table 4:** Pharmacokinetic parameters of *β*-asarone after oral administration in mice of each group.

Parameters	B1	A1B1	B	AB
C_max_ (mg/l)	0.13 ± 0.12	0.37 ± 023	0.05 ± 0.05	0.14 ± 0.07
T_max_ (h)	1.72 ± 2.09	0.20 ± 0.14	0.49 ± 0.40	0.22 ± 0.07
t_1/2z_ (h)	4.31 ± 2.52	8.34 ± 6.15	7.84 ± 4.69	6.18 ± 4.23
AUC (0-t) (mg/l^∗^h)	0.42 ± 0.29	1.06 ± 0.53	0.15 ± 0.04	0.73 ± 0.19
AUC (0-∞) (mg/l^∗^h)	0.45 ± 0.31	1.29 ± 0.56	0.17 ± 0.05	0.88 ± 0.36
V_Z/F_ (l/kg)	274.59 ± 221.19	149.30 ± 88.20	24177.28 ± 19044.13	4554.28 ± 1910.76
CL_Z/F_ (l/h/kg)	49.74 ± 33.49	14.11 ± 7.18	3098.68 ± 1168.18	624.52 ± 229.15

## Data Availability

The original contributions presented in the study are included in the article/supplementary materials; further inquiries can be directed to the corresponding author.

## Abbreviations

GFAP: Glial fibrillary acidic protein
HE: Hematoxylin-eosin
IHC: Immunohistochemical staining
IS: Ischemic stroke
MCAO: Middle cerebral artery occlusion
MJCPF: Mi-Jian-Chang-Pu formula
NF-*κ*B: Nuclear factor-*κ*B
PCA: Principal component analysis
PLS-DA: Partial least squares discriminant analysis
QC: Quality control
TCA: Tricarboxylic acid cycle
TCM: Traditional Chinese medicine
TTC: 2,3,5-triphenylte triazolium chloride
UHPLC-QTOF-MS: Ultrahigh performance liquid chromatography quadrupole-time-of-flight mass spectrometry
VEGF-c: Vascular endothelial growth factor
VIP: Variable importance for the projections.

## Supplementary Materials

Supplementary 1 Supplementary File S1: metabolic experiment.

Supplementary 2 Supplementary File S2: pharmacokinetic experiment.

Supplementary 3 Supplementary File S3: the relative content of significantly differential metabolites in each group.

## References

[1] Paul S., Candelario-Jalil E. (2021). Emerging neuroprotective strategies for the treatment of ischemic stroke: an overview of clinical and preclinical studies. *Experimental Neurology*.

[2] Wang Y., Li Z., Gu H. (2020). China stroke statistics 2019: a report from the National Center for Healthcare Quality Management in Neurological Diseases, China National Clinical Research Center for neurological diseases, the Chinese Stroke Association, National Center for Chronic and Non-Communicable Disease Control and Prevention, Chinese Center for Disease Control and Prevention and Institute for Global Neuroscience and Stroke Collaborations. *Stroke and Vascular Neurology*.

[3] Maida C. D., Norrito R. L., Daidone M., Tuttolomondo A., Pinto A. (2020). Neuroinflammatory mechanisms in ischemic stroke: focus on cardioembolic stroke, background, and therapeutic approaches. *International Journal of Molecular Sciences*.

[4] Zhang X. Y., Shen X. Z., Dong J. L. (2019). Inhibition of reactive astrocytes with fluorocitrate ameliorates learning and memory impairment through upregulating Crtc1 and synaptophysin in ischemic stroke rats. *Cellular and Molecular Neurobiology*.

[5] de Souza Queiroz J., Bazán P. R., Batista A. X., Martin M. D. G. M., Miotto E. C., de Medeiros Rimkus C. (2022). White matter microstructural damage in chronic ischemic stroke affecting the left inferior frontal gyrus: association with cognitive functions. *Clinical Neurology and Neurosurgery*.

[6] Amani H., Mostafavi E., Alebouyeh M. R. (2019). Would colloidal gold nanocarriers present an effective diagnosis or treatment for ischemic stroke. *International Journal of Nanomedicine*.

[7] Hsu W., Shen Y., Shiao Y. (2019). Combined proteomic and metabolomic analyses of cerebrospinal fluid from mice with ischemic stroke reveals the effects of a Buyang Huanwu decoction in neurodegenerative disease. *PLoS One*.

[8] Feher G., Gurdan Z., Gombos K. (2020). Early seizures after ischemic stroke: focus on thrombolysis. *CNS Spectrums*.

[9] Zhang W. W., Xu F., Wang D., Ye J., Cai S.-Q. (2018). Buyang Huanwu decoction ameliorates ischemic stroke by modulating multiple targets with multiple components: in vitro evidences. *Chinese Journal of Natural Medicines*.

[10] She Y., Shao L., Zhang Y. (2019). Neuroprotective effect of glycosides in Buyang Huanwu decoction on pyroptosis following cerebral ischemia-reperfusion injury in rats. *Journal of Ethnopharmacology*.

[11] Liu J. X., Li J. S., Niu Y., Yu W., Hei C. C., Liu H. X. (2011). Effects of Zha-Li-nu-Si decoction and Mi-Jian-Chang-Pu decoction on neuron injury in cerebral ischemia rats. *Journal of Ningxia Medical University*.

[12] Wang F. (2018). *Effects of hui medicine Mijian Changpu prescription on neural function and synaptic remodeling of hippocampal neurons in rats after cerebral ischemia reperfusion*.

[13] Yang Y. X., Chen Y. T., Zhou X. J., Hong C. L., Li C. Y., Guo J. Y. (2013). Beta-Asarone, a major component of Acorus Tatarinowii Schott, attenuates focal cerebral ischemia induced by middle cerebral artery occlusion in rats. *BMC Complementary and Alternative Medicine*.

[14] Liu F. Z., Zhao Q., Liu S. X. (2020). Revealing the pharmacological mechanism of Acorus Tatarinowii in the treatment of ischemic stroke based on network pharmacology. *Evidence-Based Complementary and Alternative Medicine*.

[15] Sadeghnia H. R., Shaterzadeh H., Forouzanfar F., Hosseinzadeh H. (2017). Neuroprotective effect of safranal, an active ingredient of crocus Sativus, in a rat model of transient cerebral ischemia. *Folia Neuropathologica*.

[16] Gudarzi S., Jafari M., Jahromi G. P., Eshrati R., Asadollahi M., Nikdokht P. (2022). Evaluation of modulatory effects of saffron (crocus Sativus L.) aqueous extract on oxidative stress in ischemic stroke patients: a randomized clinical trial. *Nutritional Neuroscience*.

[17] Liu J. J., Yang L. L., Niu Y. (2022). Potential therapeutic effects of Mi-Jian-Chang-Pu decoction on neurochemical and metabolic changes of cerebral ischemia-reperfusion injury in rats. *Oxidative Medicine and Cellular Longevity*.

[18] Nagasawa H., Kogure K. (1989). Correlation between cerebral blood flow and histologic changes in a new rat model of middle cerebral artery occlusion. *Stroke*.

[19] Wang L. D., Ji X. M., Kang D. Z. (2021). Brief Report on Stroke Center in China, 2020. *Chinese Journal of Cerebrovascular Diseases*.

[20] Geiseler S., Morland C. (2018). The Janus face of VEGF in stroke. *International Journal of Molecular Sciences*.

[21] Chen B., Wang G. X., Li W. (2016). Memantine attenuates cell apoptosis by suppressing the Calpain-Caspase-3 pathway in an experimental model of ischemic stroke. *Experimental Cell Research*.

[22] Wang F., Ji S. L., Wang M. X. (2020). HMGB1 promoted P-glycoprotein at the blood-brain barrier in MCAO rats via Tlr4/NF-*κ*B signaling pathway. *European Journal of Pharmacology*.

[23] Fan W. Y., Dai Y. Q., Xu H. C. (2014). Caspase-3 modulates regenerative response after stroke. *Stem Cells*.

[24] Simmons L. J., Surles-Zeigler M. C., Li Y. G., Ford G. D., Newman G. D., Ford B. D. (2016). Regulation of inflammatory responses by neuregulin-1 in brain ischemia and microglial cells in vitro involves the NF-kappa B pathway. *Journal of Neuroinflammation*.

[25] Yan L., Zhu T. (2019). Effects of Rosuvastatin on neuronal apoptosis in cerebral ischemic stroke rats via Sirt1/Nf-kappa B signaling pathway. *European Review for Medical and Pharmacological Sciences*.

[26] Ipata P. L., Camici M., Micheli V., Tozzi G. M. (2011). Metabolic network of nucleosides in the brain. *Current Topics in Medicinal Chemistry*.

[27] Lin X., Lécuyer L., Liu X. (2021). Plasma metabolomics for discovery of early metabolic markers of prostate cancer based on ultra-high-performance liquid chromatography-high resolution mass spectrometry. *Cancers*.

[28] Yang M. X., Wang S., Hao F. H., Li Y., Tang H., Shi X. (2012). NMR analysis of the rat neurochemical changes induced by middle cerebral artery occlusion. *Talanta*.

[29] Licari C., Tenori L., Giusti B. (2021). Analysis of metabolite and lipid association networks reveals molecular mechanisms associated with 3-month mortality and poor functional outcomes in patients with acute ischemic stroke after thrombolytic treatment with recombinant tissue plasminogen activator. *Journal of Proteome Research*.

[30] Jensen N. J., Wodschow H. Z., Nilsson M., Rungby J. (2020). Effects of ketone bodies on brain metabolism and function in neurodegenerative diseases. *International Journal of Molecular Sciences*.

[31] Moghadam R. A., Molazadeh L., Suha Z. J., Naghizadeh-Baghi A., Mohajeri M., Nemati A. (2022). Glutamine supplementation can reduce some atherosclerosis markers after exhaustive exercise in young healthy males. *Nutrition*.

[32] Gupta S., Sharma U., Jagannathan N. R., Gupta Y. K. (2020). ^1^H NMR metabolomic profiling elucidated attenuation of neurometabolic alterations by Lercanidipine in MCAO model in rats. *Journal of Pharmacy and Pharmacology*.

[33] Sun H., Dong W., Zhang A. H., Wang W., Wang X. (2012). Pharmacokinetics study of multiple components absorbed in rat plasma after oral administration of Stemonae Radix using ultra-performance liquid-chromatography/mass spectrometry with automated Metabolynx software analysis. *Journal of Separation Science*.

