# Correction to “The Combination of Individual Herb of Mi‐Jian‐Chang‐Pu Formula Exerts a Synergistic Effect in the Treatment of Ischemic Stroke in Rats”

**DOI:** 10.1155/omcl/9795781

**Published:** 2026-03-14

F. Lu, X. Su, J. Liu, R. Zong, S. Ding, L. Yang, J. Liu, G. Wilson, L. Li, Y. Yang, X. Wang, W. Wang, and X. Ma, “The Combination of Individual Herb of Mi‐Jian‐Chang‐Pu Formula Exerts a Synergistic Effect in the Treatment of Ischemic Stroke in Rats” *Oxidative Medicine and Cellular Longevity*, vol. 2022 (2022) https://doi.org/10.1155/2022/9365760.

In the article titled “The Combination of Individual Herb of Mi‐Jian‐Chang‐Pu Formula Exerts a Synergistic Effect in the Treatment of Ischemic Stroke in Rats,” there was an error in Figure 6. More specifically, an incorrect image was used to represent the ICH staining of the VEGF‐B1 group. This error was introduced by the authors during figure assembly, and Figure 6 should be corrected as follows:

**Figure 6:**
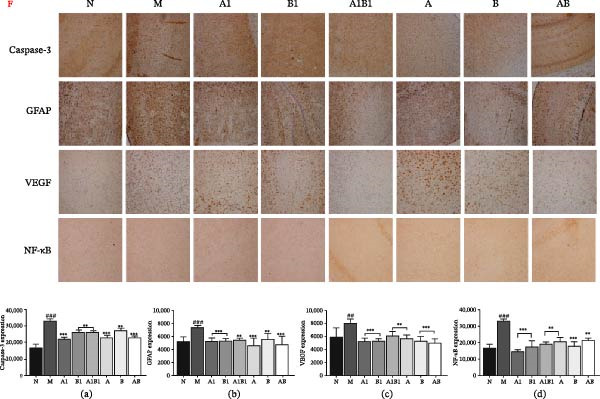
Effect of drug on protein levels of caspase3, GFAP, VEGF, and NF‐κB in cortex of MCAO rats. Drug treatment significantly reversed protein expression. (F) Brain coronal sections were selected for ICH staining; (a–d) were the statistical results of protein expression of caspase 3, GFAP, VEGF, and NF‐κB, respectively.  ^∗∗^ *p*  < 0.01,  ^∗∗∗^ *p*  < 0.001, as compared to M group; ^##^ *p*  < 0.01, ^###^ *p*  < 0.001, as compared to N group.

We apologize for this error.

