# Supplementary material for: The Combination of Individual Herb of Mi-Jian-Chang-Pu Formula Exerts a Synergistic Effect in the Treatment of Ischemic Stroke in Rats
Source: Oxid Med Cell Longev. 2022 Oct 18;2022:9365760. doi: 10.1155/2022/9365760 (PMC9597002; doi:10.1155/2022/9365760)
Supplement: Supplementary 1 — Supplementary File S1: metabolic experiment. [file 9365760.f1.docx]

**Supplementary File S1--Metabolic experiment**

**1. Methods**

1.1 Preparation of internal and mixed standard solutions

An internal standard solution was prepared by using [astragaloside Ⅳ](https://pubmed.ncbi.nlm.nih.gov/29926836) (10.6μg/ml) dissolved in methanol.

A mixed standard stock solution containing 10 standard references was prepared, including tryptophan (3.6 μg/ml), pantothenic acid (4.64 μg/ml), stearic acid (2.18 μg/ml), 3-methylindole (2.2 μg/ml), butyl 4-hydroxybenzoate (4.6 μg/ml), vedaprofen (7.6 μg/ml), 1-myristoyl-2-hydroxy-sn-glycerol-3-phosphocholine (2.5 μg/ml), salsolinol (6.4 μg/ml), methyl jasmonate (6.8 μg/ml) and isofraxidin (9.2 μg/ml), then this standard stock solution (H) was diluted 2 and 4 times as medium (M) and low (L) concentrations, respectively.

1.2 Extraction recovery and matrix effect

The extraction recovery was investigated by comparing the peak area of the blank serum samples with analytes and internal standard spiked before and after extraction, as showed in Eq. (1); and the matrix effect was investigated by comparing the peak areas of the processed blank serum samples (analytes and internal standard solution were added) after extraction with the corresponding standard solutions, as presented in Eq. (2)

Extraction recovery=A_p_/A_x_×100% Eq. (1)

Matrix effect=A_p_/A_s_×100% Eq. (2)

A_p_: 10 μl of high (H), medium (M), and low (L) concentration of standard solution and 150 μl of internal standard acetonitrile were added to 30 μl of blank serum, respectively, then pre-processed by sample pretreatment method and injected to obtain the peak area A_p_.

A_x_: 150 μl of internal standard was added to 30 μl of blank serum. After pre-treatment according to the preparation of the sample solution, 10 μl of high, medium and low concentration standard solution was added respectively, and the peak area A_x_ was obtained by injection.

A_s_: 10 μl of high, medium and low concentration of standard solution and 150 μl of internal standard solution were added into 30 μl of methanol solvent, then pre-processed by sample pretreatment method and injected to obtain the peak area A_s_.

1.3 Precision test

The precision was assessed by determining the peak area of six replicates of QC samples on consecutive three days. The precision was expressed as relative standard deviation (*RSD*) and required within ±15%.

1.5 Stability test

Before testing the sample solution, QC (n=6) were continuously monitored. A QC sample was tested after every 10 samples, and the data obtained was analyzed by using PCA to investigate the stability of the system.

The stability of analytes in serum and brain was assessed by analyzing peak area of QC samples. The short-term stability was evaluated by analyzing samples stored on the bench top at room temperature (25℃) for 12 h. The long-term stability was evaluated by analyzing samples after stored at -20℃ for 4 weeks. The freeze-thaw stability was evaluated by analyzing samples after three freeze-thaw cycles (-80℃ to 25℃). Post-preparative stability was assessed by analyzing samples after stored in the autosampler at 4℃ for 24 h.

**2. Results**

By using serum and brain QC samples, the stability of the entire analysis process was monitored, and the conditions of UHPLC-QTOF-MS/MS was optimized. Data acquisition in both positive and negative ion modes indicated a good repeatability of QC samples (**Supplementary Figure S1**), implied the stability of the apparatus.

The results of stability and precision are summarized in **Supplementary** **Table S1** and **Supplementary** **Table S2**. No obvious degradation was found in the short-term, long-term and three freeze-thaw cycles experiments as well as in the post-pretreatment samples, indicating that the analytes were stable in the serum/brain. The *RSD* was no more than 14.61%. All the results indicated that the method in this study was acceptable for its good stability and precision.

The results of the extraction recovery and matrix effect of samples at three concentration levels were shown in **Supplementary** **Table S3**. The mean extraction recoveries of the all the analytes were between 84.85% and 113.27%, and the mean matrix effects were ranged from 92.19% to 117.09%. All the results indicated that the sample pretreatment was appropriate to obtain stable and high extraction recovery and no obvious endogenous interference existed in the serum.


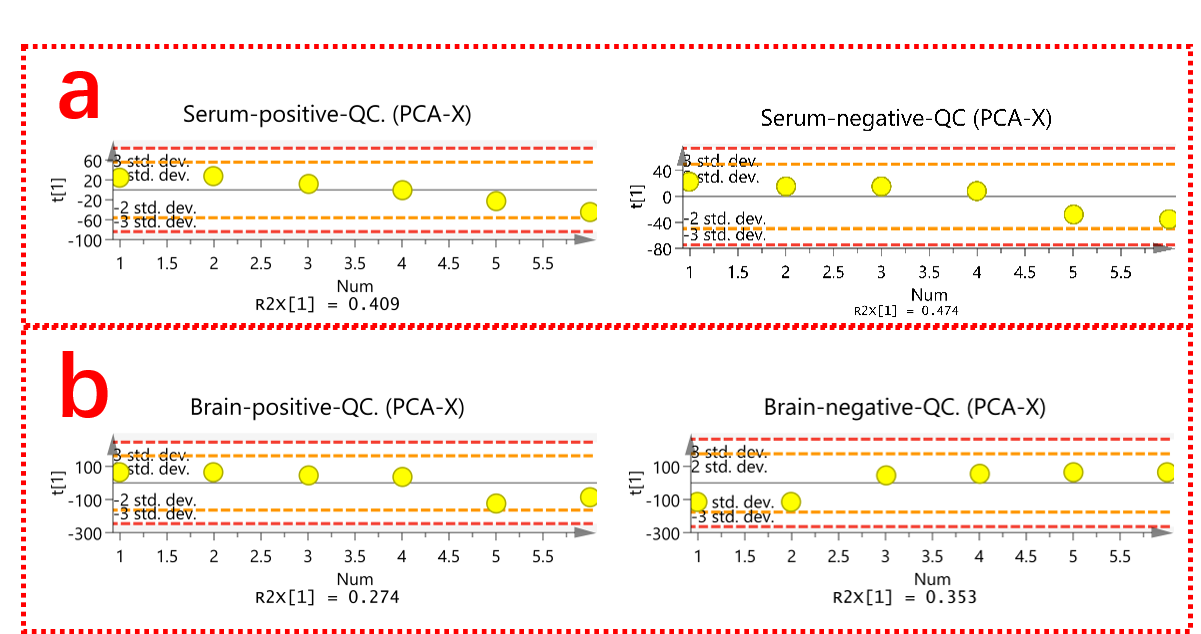


**Supplementary Figure S1** Projection results of the first principal component of the PCA model of the QC sample in positive and negative ion modes. a: serum samples in positive/negative. b: brain samples in positive/negative.

**Supplementary Table S1** Results of stability and precision of serum in positive and negative ion modes

| Model | m/z | Rt | Intraday stability (*RSD* %) | Long-term stability (*RSD* %) | Freeze-thaw stability (*RSD* %) | Post-preparation stability (*RSD* %) | Precision (*RSD* %) |
| --- | --- | --- | --- | --- | --- | --- | --- |
| Positive ion | 132.0808 | 0.712 | 1.27 | 9.37 | 3.58 | 2.15 | 1.69 |
|  | 180.1019 | 1.474 | 14.15 | 12.71 | 4.92 | 4.57 | 13.54 |
|  | 205.0972 | 2.186 | 9.13 | 4.02 | 3.46 | 11.26 | 3.41 |
|  | 195.1016 | 3.313 | 6.21 | 1.92 | 0.97 | 6.05 | 1.57 |
|  | 223.0601 | 3.313 | 6.22 | 1.61 | 0.97 | 6.05 | 1.57 |
|  | 225.1485 | 7.057 | 6.08 | 11.27 | 1.67 | 7.24 | 11.35 |
|  | 469.3163 | 7.322 | 4.08 | 3.01 | 0.81 | 9.15 | 2.47 |
|  | 283.1693 | 14.429 | 4.39 | 6.51 | 12.23 | 7.28 | 3.82 |
|  | 285.2788 | 16.400 | 5.75 | 6.33 | 7.14 | 7.65 | 13.58 |
|  | 271.2632 | 18.272 | 4.47 | 4.19 | 9.16 | 13.71 | 7.59 |
| Negative ion | 130.0602 | 0.610 | 8.12 | 8.42 | 1.66 | 9.63 | 1.19 |
|  | 203.0826 | 2.184 | 7.49 | 9.03 | 5.09 | 2.65 | 4.28 |
|  | 178.0874 | 3.310 | 5.39 | 2.85 | 1.81 | 2.35 | 2.19 |
|  | 193.0870 | 6.607 | 3.31 | 4.46 | 3.92 | 2.54 | 2.91 |
|  | 221.0455 | 6.706 | 10.73 | 10.29 | 5.22 | 8.85 | 4.86 |
|  | 223.1340 | 6.706 | 10.32 | 10.29 | 5.92 | 8.55 | 4.37 |
|  | 467.3017 | 9.423 | 6.33 | 10.77 | 10.98 | 11.89 | 8.91 |
|  | 269.2486 | 10.417 | 5.55 | 5.53 | 5.74 | 8.50 | 8.26 |
|  | 283.2643 | 10.610 | 11.41 | 8.79 | 7.11 | 11.19 | 12.51 |
|  | 281.1547 | 16.547 | 13.82 | 13.89 | 13.36 | 12.68 | 4.32 |

**Supplementary Table S2** Results of stability and precision of brain in positive and negative ion modes

| Model | m/z | Rt (min) | Intraday stability (*RSD* %) | Post-preparation stability (*RSD* %) | Precision (*RSD* %) |
| --- | --- | --- | --- | --- | --- |
| Positive ion | 137.0458 | 0.852 | 0.62 | 10.07 | 11.61 |
|  | 188.0706 | 2.508 | 1.33 | 10.33 | 10.74 |
|  | 415.2110 | 10.476 | 2.62 | 12.67 | 14.61 |
|  | 312.3624 | 14.635 | 4.68 | 2.17 | 6.17 |
|  | 663.4528 | 17.527 | 2.64 | 3.74 | 9.56 |
| Negative ion | 112.9587 | 0.628 | 0.56 | 2.81 | 10.91 |
|  | 267.0733 | 1.721 | 0.64 | 3.89 | 6.63 |
|  | 293.1762 | 9.292 | 3.17 | 3.87 | 10.52 |
|  | 524.2782 | 14.543 | 2.77 | 7.47 | 13.52 |
|  | 303.2331 | 16.962 | 5.59 | 14.43 | 9.16 |

**Supplementary Table S3** Results of matrix effect and extraction recovery of serum in positive and negative ion modes

| Model | m/z | Rt | Extraction recovery | | | matrix effects | | |
| --- | --- | --- | --- | --- | --- | --- | --- | --- |
|  |  |  | H (%) | M (%) | L (%) | H (%) | M (%) | L (%) |
| Positive  ion | 132.0808 | 0.712 | 113.27 | 100.84 | 98.65 | 105.25 | 100.41 | 101.26 |
|  | 180.1019 | 1.474 | 101.45 | 101.45 | 97.63 | 105.9 | 94.02 | 110.99 |
|  | 205.0972 | 2.186 | 111.06 | 100.67 | 93.39 | 112.35 | 98.15 | 117.09 |
|  | 195.1016 | 3.313 | 103.71 | 97.61 | 96.11 | 110.97 | 98.45 | 96.02 |
|  | 223.0601 | 3.313 | 103.06 | 96.61 | 96.39 | 110.05 | 92.19 | 98.05 |
|  | 225.1485 | 7.057 | 98.41 | 86.56 | 102.84 | 102.38 | 99.53 | 102.81 |
|  | 469.3163 | 7.322 | 88.51 | 91.07 | 89.34 | 96.56 | 105.04 | 110.04 |
|  | 283.1693 | 14.429 | 95.93 | 100.46 | 104.16 | 104.82 | 104.62 | 106.21 |
|  | 285.2788 | 16.400 | 93.28 | 108.14 | 98.19 | 104.95 | 101.11 | 98.39 |
|  | 271.2632 | 18.272 | 106.88 | 101.08 | 104.97 | 100.27 | 95.32 | 99.75 |
| Negative ion | 130.0602 | 0.610 | 98.73 | 108.26 | 103.42 | 115.38 | 112.97 | 113.36 |
|  | 203.0826 | 2.184 | 106.73 | 98.37 | 101.94 | 104.82 | 107.27 | 105.96 |
|  | 178.0874 | 3.310 | 104.72 | 99.26 | 90.95 | 94.56 | 108.35 | 103.21 |
|  | 193.0870 | 6.607 | 95.58 | 100.31 | 102.86 | 98.53 | 98.28 | 98.53 |
|  | 221.0455 | 6.706 | 108.76 | 95.31 | 99.51 | 106.96 | 99.14 | 103.79 |
|  | 223.1340 | 6.706 | 108.12 | 95.18 | 95.51 | 112.18 | 111.17 | 105.45 |
|  | 467.3017 | 9.423 | 102.13 | 96.14 | 100.39 | 101.29 | 107.75 | 90.62 |
|  | 269.2486 | 10.417 | 101.85 | 118.87 | 104.25 | 101.12 | 99.06 | 102.59 |
|  | 283.2643 | 10.610 | 104.28 | 84.85 | 98.43 | 105.12 | 104.16 | 101.74 |
|  | 281.1547 | 16.547 | 101.62 | 97.62 | 93.94 | 107.44 | 97.02 | 102.06 |
