# Supplementary material for: The Combination of Individual Herb of Mi-Jian-Chang-Pu Formula Exerts a Synergistic Effect in the Treatment of Ischemic Stroke in Rats
Source: Oxid Med Cell Longev. 2022 Oct 18;2022:9365760. doi: 10.1155/2022/9365760 (PMC9597002; doi:10.1155/2022/9365760)
Supplement: Supplementary 2 — Supplementary File S2: pharmacokinetic experiment. [file 9365760.f2.docx]

**Supplementary File S2—Pharmacokinetic experiment**

**1. Methods**

1.1 Preparation of internal and mixed standard solutions

Internal standard solution was prepared by employing matrine dissolved in methanol (10 μg/ml).

A mixed stock standard solution containing 8.76 μg/ml of crocin I and 8.04 μg/ml of β-asarone was prepared using acetonitrile-water (1:1) as solvent. Then this mixed standard solution was diluted 2 and 4 times as medium and low concentrations, respectively.

1.2 Linear experiment

Serial standard solutions of crocin I and β-asarone were added in blank plasma. Five levels of the calibration curve were determined. The ratio of peak area of internal standard and sample was Y, and the concentration was X, respectively. The linear regression equation was obtained by regression calculation using the least-square linear regression method. The final concentrations of crocin I in plasma were 2,5,10,20,40 ng/ml and β-asarone were 2,5,10,15,20 ng/ml.

1.3 Precision test

The precision was assessed by determining the peak area of six replicates of QC samples on consecutive three days. The precision was expressed as relative standard deviation (*RSD*) and required within ±15%.

1.4 Stability test

The stability of analytes in plasma and brain was assessed by analyzing peak area of QC samples. The short-term stability was evaluated by analyzing samples stored on the bench top at room temperature (25℃) for 12 h. The long-term stability was evaluated by analyzing samples after stored at -20℃ for 4 weeks. The freeze-thaw stability was evaluated by analyzing samples after three freeze-thaw cycles (-80℃ to 25℃). Post-preparative stability was assessed by analyzing samples after stored in the autosampler at 4℃ for 24 h.

1.5 Extraction recovery and matrix effect

The extraction recovery was investigated by comparing the peak area of the blank plasma samples with analytes and internal standard spiked before and after extraction, as showed in Eq. (1); and the matrix effect was investigated by comparing the peak areas of the processed blank plasma samples (analytes and internal standard solution were added) after extraction with the corresponding standard solutions, as presented in Eq. (2)

Extraction recovery=A_p_/A_x_×100% Eq. (1)

Matrix effect=A_p_/A_s_×100% Eq. (2)

A_p_: 10 μl of high (H), medium (M), and low (L) concentration of standard solution and 150 μl of internal standard acetonitrile were added to 30 μl of blank plasma, respectively, then pre-processed by sample pretreatment method and injected to obtain the peak area A_p_.

A_x_: 150 μl of internal standard was added to 30 μl of blank plasma. After pre-treatment according to the preparation of the sample solution, 10 μl of high, medium and low concentration standard solution was added respectively, and the peak area A_x_ was obtained by injection.

A_s_: 10 μl of high, medium and low concentration of standard solution and 150 μl of internal standard solution were added into 30 μl of methanol solvent, then pre-processed by sample pretreatment method and injected to obtain the peak area A_s_.

**2. Results**

The calibration curve standards were prepared and analyzed at 5 different concentrations from 2 to 40 ng/ml for crocin I and 2 to 20 ng/ml for β-asarone. As is shown in **Supplementary Figure S1**, the calibration curve for crocin I and β-asarone demonstrated acceptable linearity (mean correlation coefficient r > 0.99) in this concentration. The linear regression equation of crocin Ⅰ was y=-0.22x+11.94, β-asarone was y=-0.36x＋11.40.


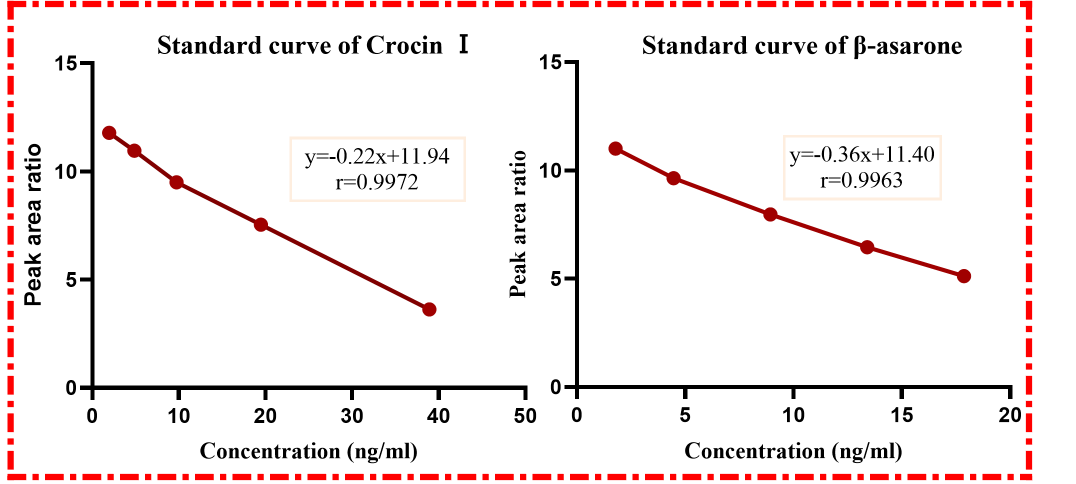


**Supplementary Figure S1.** Standard curve of crocin Ⅰ and β-asarone

The results of stability, precision and repeatability are summarized in **Supplementary** **Table S1**. No obvious degradation was found in the short-term, long-term and three freeze-thaw cycles experiments as well as in the post-pretreatment samples, indicating that the analytes were stable in the plasma. The *RSD* was no more than 12.77%. All the results indicated that the method in this study was acceptable for its good stability, repeatability and precision.

The results of the extraction recovery and matrix effect of samples at three concentration levels were shown in **Supplementary** **Table S2**. The mean extraction recoveries of the all the analytes were between 94.17% to 113.09%, and the mean matrix effects were ranged from 96.29% to 107.89%. All the results indicated that the sample pretreatment was appropriate to obtain stable and high extraction recovery and no obvious endogenous interference existed in the plasma.

**Supplementary Table S1** Results of stability, repeatability and precision of plasma sample

| sample | Rt  (min) | stability | | | | Precision (RSD%) | Repeatabiliy (RSD%) |
| --- | --- | --- | --- | --- | --- | --- | --- |
|  |  | Intraday (RSD%) | Long-term (RSD%) | Freeze-thaw (RSD%) | Post-preparation (RSD%) |  |  |
| matrine | 0.64 | 12.01 | 7.37 | 9.46 | 8.45 | 9.38 | 10.33 |
| crocin I | 2.91 | 8.37 | 5.86 | 12.36 | 12.77 | 4.35 | 10.01 |
| β-asarone | 4.46 | 9.77 | 5.43 | 4.48 | 5.71 | 7.73 | 6.74 |

**Supplementary Table S2** Results of matrix effect and extraction recovery of plasma sample

| sample | Rt | extraction recovery | | | matrix effect | | |
| --- | --- | --- | --- | --- | --- | --- | --- |
|  |  | H (%) | M (%) | L (%) | H (%) | M (%) | L (%) |
| crocin I | 2.91 | 98.25 | 94.17 | 101.62 | 107.89 | 101.96 | 105.47 |
| β-asarone | 4.46 | 113.09 | 104.4 | 99.17 | 96.29 | 104.15 | 101.06 |
