# Supplementary material for: The Combination of Individual Herb of Mi-Jian-Chang-Pu Formula Exerts a Synergistic Effect in the Treatment of Ischemic Stroke in Rats
Source: Oxid Med Cell Longev. 2022 Oct 18;2022:9365760. doi: 10.1155/2022/9365760 (PMC9597002; doi:10.1155/2022/9365760)
Supplement: Supplementary 3 — Supplementary File S3: the relative content of significantly differential metabolites in each group. [file 9365760.f3.docx]

**Supplementary File S3** The relative content of significantly differential metabolites in in each group


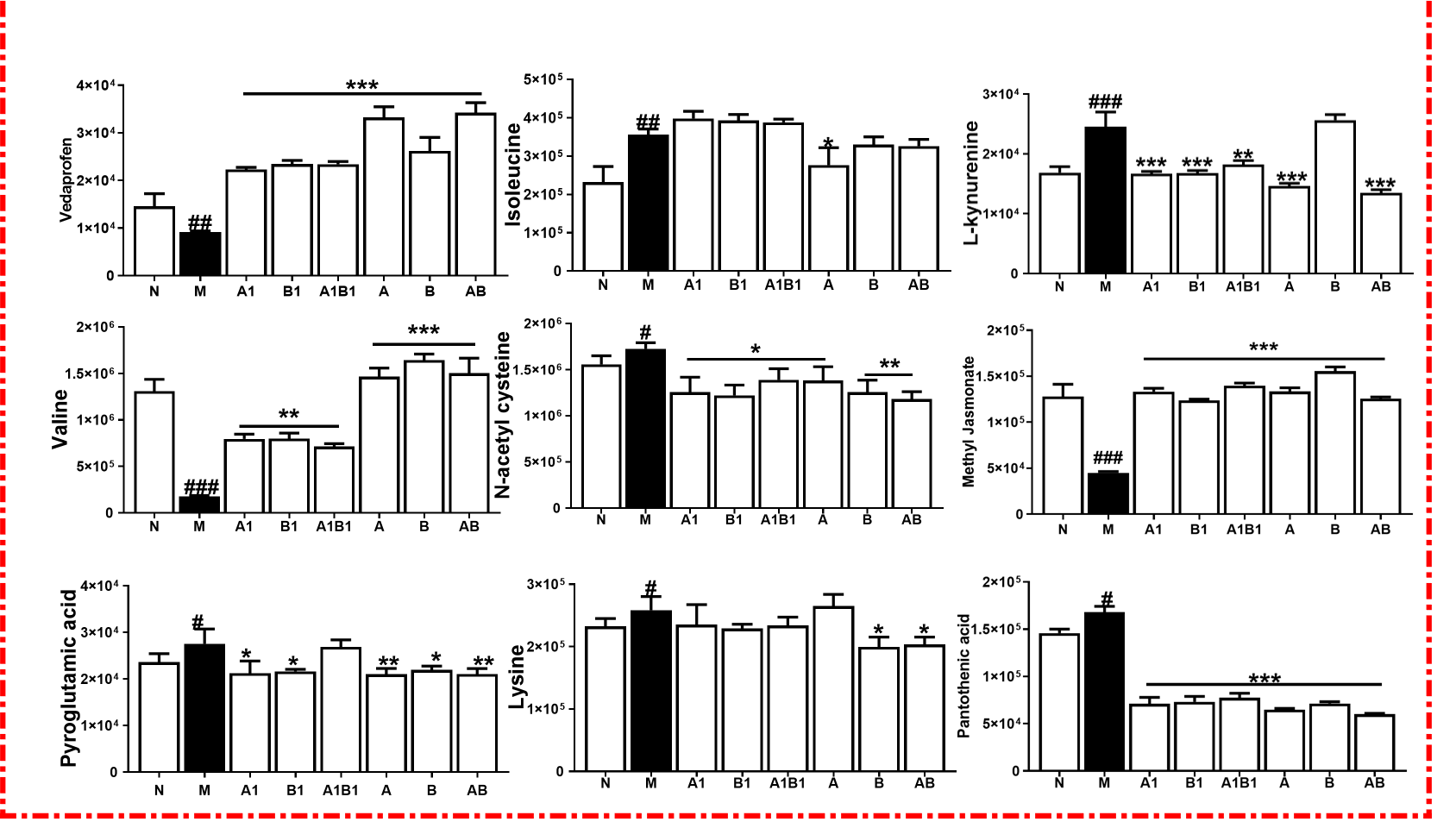

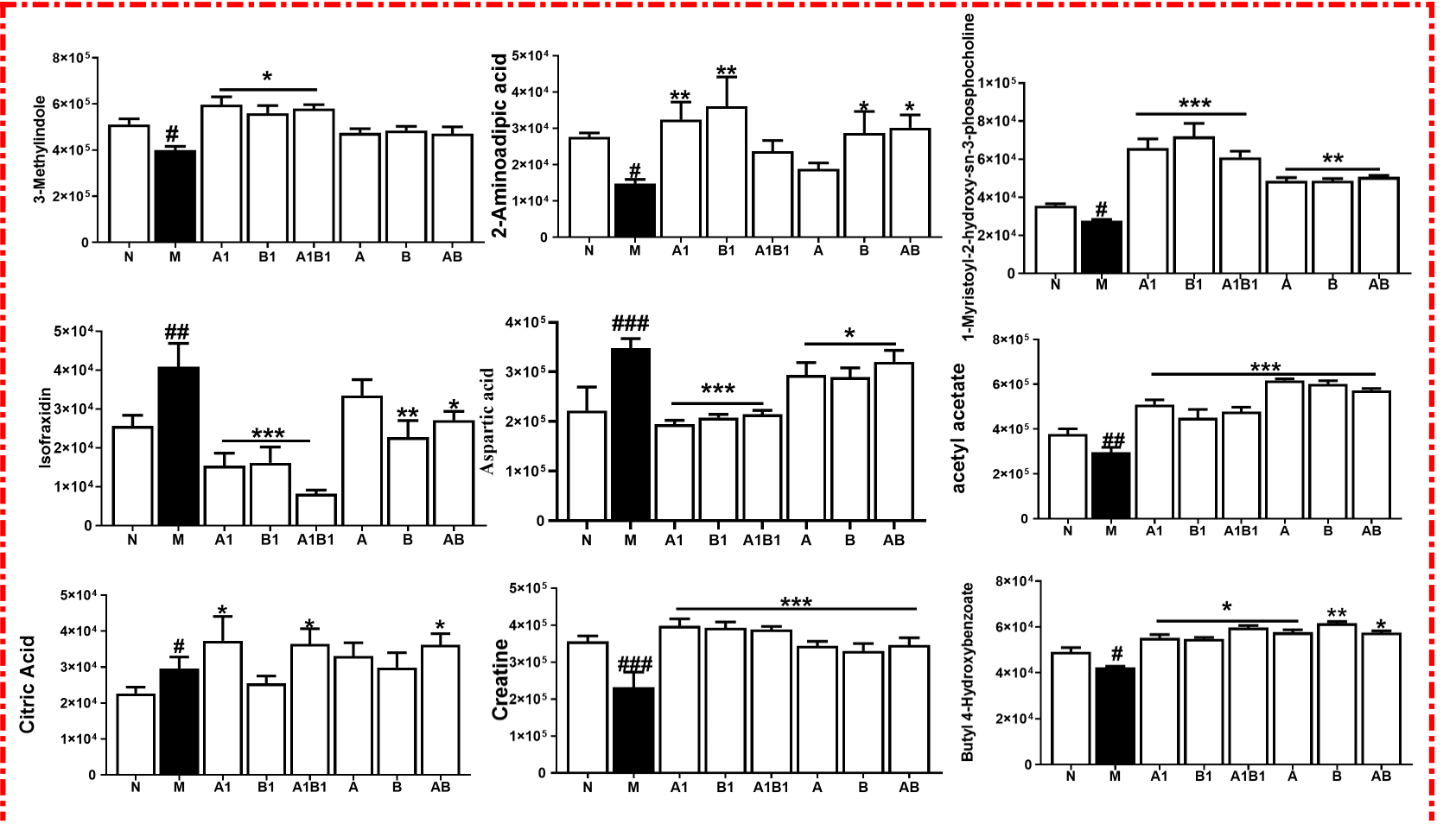


**Supplementary Figure S1** The relative content of significantly differential metabolites in positive ion mode of serum samples


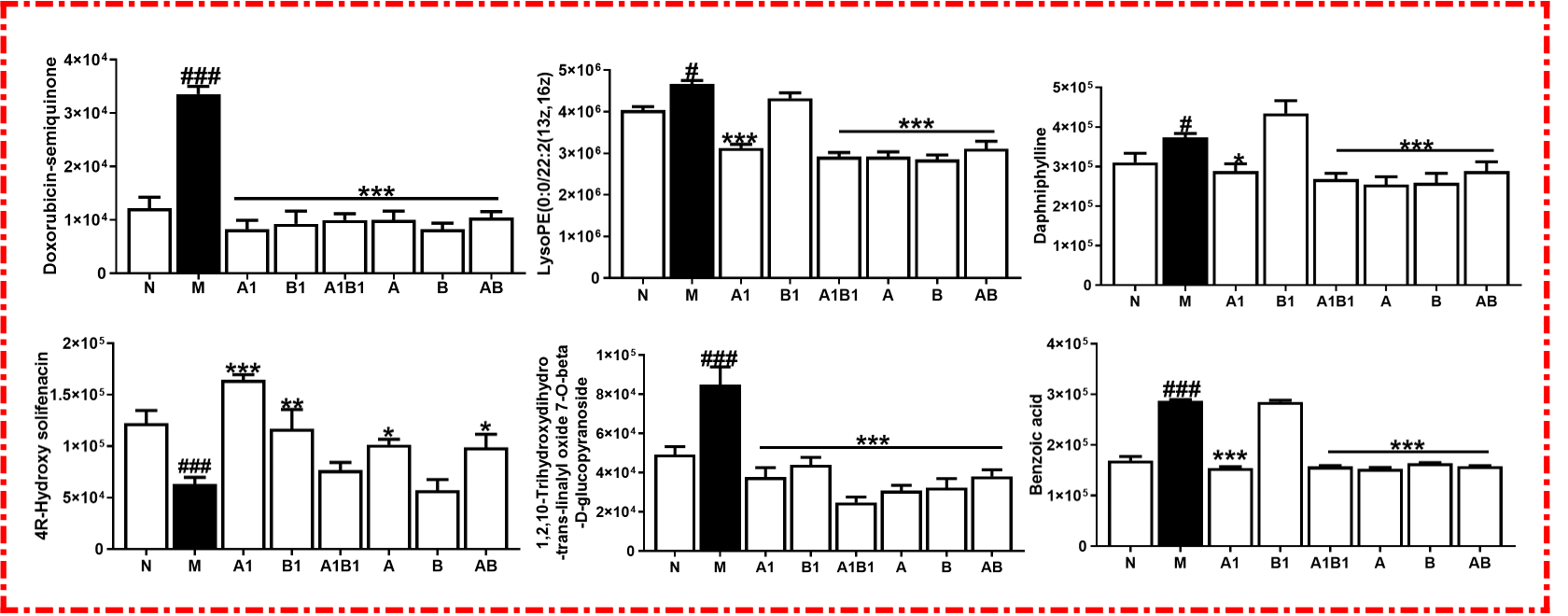


**Supplementary Figure S2** The relative content of significantly differential metabolites in negative ion mode of serum samples


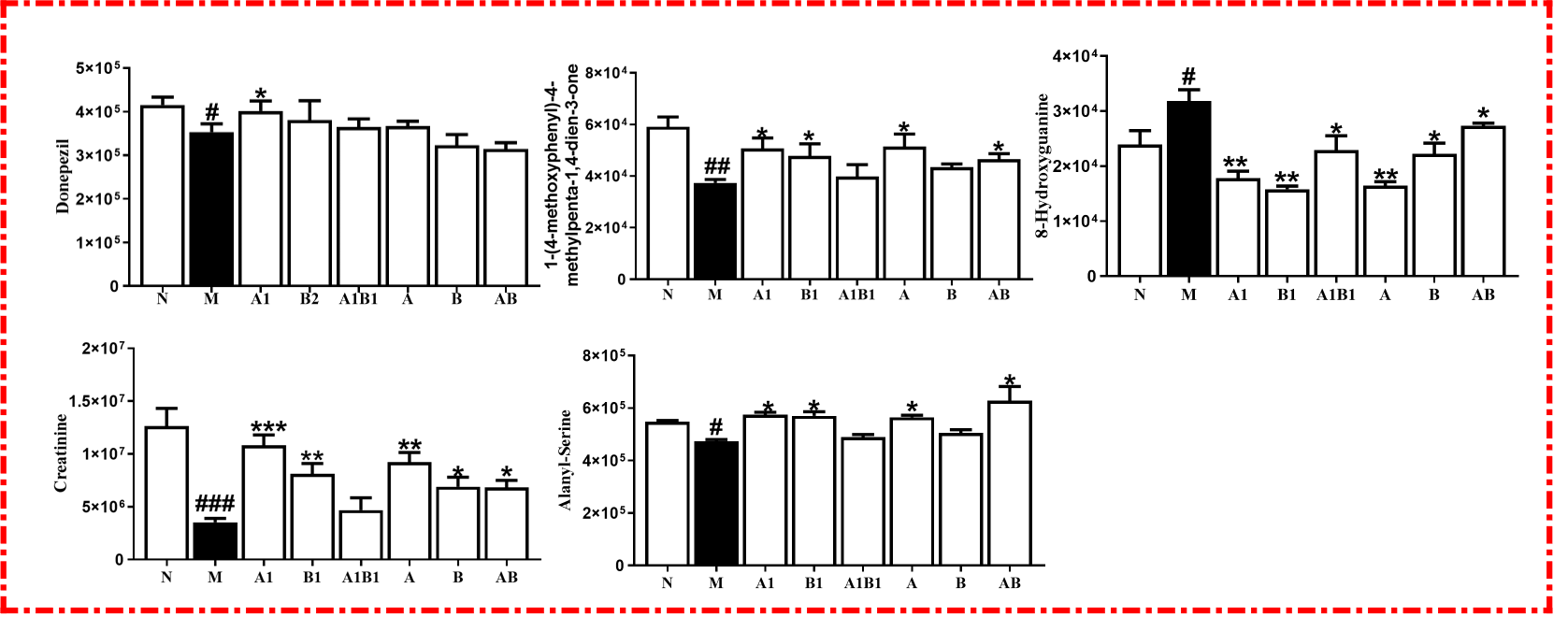


**Supplementary Figure S3** The relative content of significantly differential metabolites in positive ion mode of brain samples


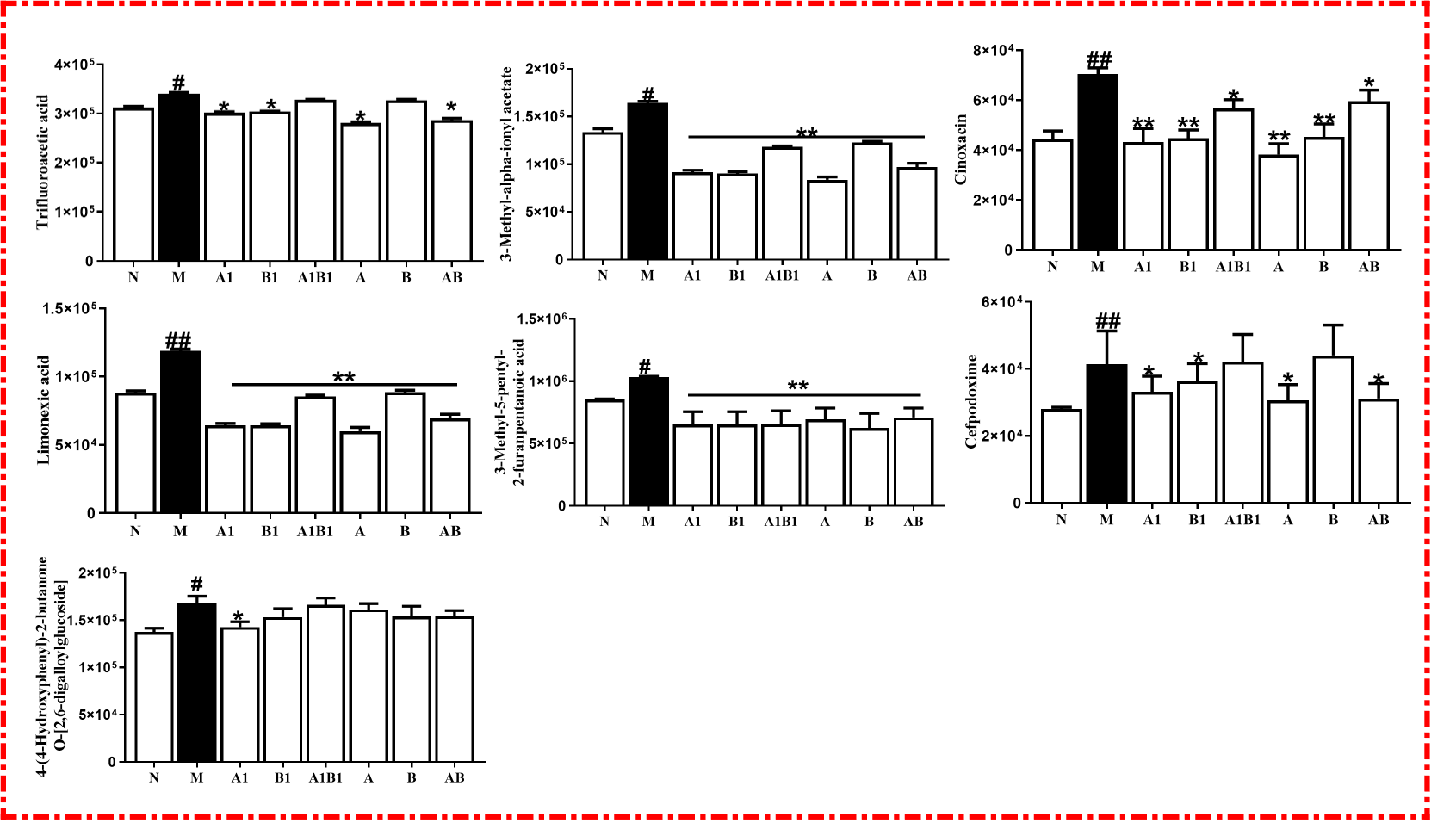


**Supplementary Figure S4** The relative content of significantly differential metabolites in negative ion mode of brain samples
